# The evolution of male–female dominance relations in primate societies

**DOI:** 10.1073/pnas.2500405122

**Published:** 2025-07-07

**Authors:** Elise Huchard, Peter M. Kappeler, Nikolaos Smit, Claudia Fichtel, Dieter Lukas

**Affiliations:** ^a^Anthropologie Evolutive, Institut des Sciences de L’Evolution de Montpellier, Université de Montpellier, CNRS, Institut de Recherche pour le Développement, Montpellier 34095, France; ^b^Behavioral Ecology and Sociobiology Unit, German Primate Center, Leibniz Institute for Primate Research, Göttingen 37077, Germany; ^c^Department of Sociobiology/Anthropology, Johann-Friedrich-Blumenbach Institute of Zoology and Anthropology, University of Göttingen, Göttingen 37077, Germany; ^d^Department of Human Behavior, Ecology and Culture, Max Planck Institute for Evolutionary Anthropology, Leipzig 04103, Germany

**Keywords:** social hierarchy, sexual conflict, primatology

## Abstract

Historically, it was widely assumed that males dominate females socially in most mammals. However, recent studies revealed significant variation within and among species, opening new possibilities to explore the extent and drivers of sex biases in dominance relations. This study uses quantitative data from 253 populations across 121 primate species to investigate the distribution of, and factors associated with, sex biases in the outcome of male–female contests. We first showed that male–female contests are common (around half of all contests) and that males win >90% of these contests in less than 20% of populations. We next tested five hypotheses to explain sex biases in dominance relations. We found that female-biased dominance primarily occurs in primate societies where females have substantial reproductive control, as in monogamous, sexually monomorphic, and arboreal species. Female-biased dominance is also frequent in societies where female–female competition is intense, as in solitary or pair-living species where females are intolerant of each other, as well as in species where females face lower reproductive costs and are philopatric. Conversely, male-biased dominance is common in polygynous, dimorphic, terrestrial, and group-living species and often relies on physical superiority. In contrast, female empowerment hinges on alternative strategies, such as leveraging reproductive control. Our study highlights that male–female dominance relationships are highly variable and identifies the traits associated with the emergence of female- versus male-biased dominance in primate evolutionary history, which may also deepen our understanding of the origins of gender roles in early human societies.

## Significance

Males were long believed to dominate females socially in most primates. Recent studies have challenged this perspective, paving the way for a more comprehensive exploration of male–female power relations. Here, we quantify and examine variation in intersexual dominance relationships across 121 primate species. We show that societies where males win nearly all aggressive encounters against females are actually rare. Evolutionarily, females became more dominant when they gained more control over reproduction, as in monogamous, monomorphic, or arboreal species, as well as when they faced more competition, as in solitary or pair-living species. Contrarily, male-biased dominance prevails in terrestrial, sexually dimorphic, and polygynous species. These results may also deepen debates about the origins of gender roles in human societies.

The first descriptions of female dominance over males in spotted hyenas (1), ring-tailed lemurs and sifakas (2) sparked considerable interest in identifying the conditions favoring the emergence of such societies (3, 4), because it was historically assumed that males are socially dominant over females in mammals (3, 5–7). Additional cases of female dominance have since been reported (7–9), including in cooperatively breeding species such as meerkats (10) and mole-rats (11), which are characterized by intense reproductive competition among females, as well as in promiscuous species where females mate with many males, such as bonobos, one of our closest living relatives (12). Despite a growing empirical record, comparative studies of sex biases in dominance relationships across mammals have been scarce, sometimes qualitative and often limited to few predictors that only characterize particular taxa (3, 5, 8, 13, 14). A broad and systematic comparative approach may therefore not only link the largely disparate fields of sexual conflict and social evolution but may also shed light on possible evolutionary origins of widespread power inequalities between genders across human societies (7, 15–19).

Investigations of sex biases in dominance across mammalian societies have been limited in several ways. First, compared to intrasexual competition, the importance and relevance of intersexual dominance hierarchies have long been downplayed because males and females were thought to compete over different resources, i.e., mates and food, respectively (20), and to rely on different mechanisms of dominance acquisition (21). Second, species were historically classified as either strictly male- or strictly female-dominant, sometimes with no firm quantitative basis, with male dominance being considered as the default state and female dominance as an exception (3, 13, 22). However, recent research has revealed that intersexual hierarchies represent a meaningful tool to quantify male–female power asymmetries, which not only vary continuously across species from complete male to complete female dominance but also exhibit flexibility within species, indicating that the relative dominance of females may often vary with age or other individual traits, as well as between contexts (22, 23). This research is based on recent progress with the definitions of core concepts, such as power, dominance, and leadership, as well as on how and why they may differ between males and females (Box 1, 15–17, 22, 24–26). In particular, resource-holding power, defined as an individual’s capacity to control access to resources and reproduction (16), is a crucial fitness determinant. It is often imposed through physical or numerical force but can also be obtained by other means, such as leverage or manipulation (24, 27). Systematic observations of which individuals win contests reveal not only how asymmetries in resource-holding power translate into stable dominance relationships (26) but also provide an opportunity to investigate the evolutionary drivers of sex biases in power in a comparative framework.

Box 1. Glossary Coercion: Strategy to influence the behavior of others using some form of physical or psychological pressure, which often involves aggression and/or threats and may incur immediate, direct costs or delayed, indirect costs for the target.

**Contest:** Any agonistic interaction involving aggressive and/or submissive acts or signals. In the literature sometimes used synonymously with conflict.
**Dominance:** Resource-holding power that is acquired and maintained using coercion.
**Intersexual hierarchy:** Ordinal ranking of males and females belonging to the same social group according to their relative dominance, established by the outcome of dyadic contests.
**Intersexual power:** Degree of control over resources and reproduction that members of one sex exert over members of the other sex.
**Leadership:** Ability of an individual to influence the behavior of others in ways that generate collective activities in various contexts, such as movement, foraging, hunting, and intergroup conflict.
**Leverage:** Bargaining asymmetry in the control over the modality of an exchange that arises between trading individuals when one possesses a desirable commodity that cannot be taken by force by others (e.g., skills, information, and under certain conditions, fertilizable eggs).
**Power:** Ability to elicit particular behaviors in others. Leadership and resource-holding power represent different dimensions of power.
**Reproductive control:** Extent to which an individual can influence the modality of its own reproduction and/or that of others (competitors and potential mates) in terms of the occurrence, timing, and frequency of matings and the number and identity of mates
**Resource-holding power:** Degree of control over resources and reproduction that one individual exerts over others, which can be acquired coercively (dominance) or by noncoercive means such as leverage.
**Win:** Bouts in which one animal issued only aggressive or nonagonistic behavior while its opponent expressed only submissive behavior are termed wins or decided conflicts in favor of the former individual.

Here, we use published data from 253 studies of 121 species representing all main lineages of the order Primates to perform comparative phylogenetically controlled analyses and i) determine the relative frequency of intra- versus intersexual contests across species, ii) investigate the taxonomic distribution of sex biases in winning these contests across species, and the extent of their variation within species, and iii) assess support for five nonmutually exclusive hypotheses that have been proposed in the literature to explain the evolutionary origin and maintenance of sex biases in dominance across primate societies.

The “reproductive control hypothesis”, in the context of sexual conflict, proposes that males and females are engaged in an evolutionary arms race over reproductive control (Box 1, 28), which shapes variation in mating systems and intersexual dominance (22). When females control reproduction, they can acquire intersexual power via leverage because males must negotiate access to sex instead of using coercion, presumably hampering males’ tendencies to initiate intersexual contests (13, 24). Therefore, female strategies to circumvent male sexual monopolization, such as manipulating the reliability of fertility signals or the duration of sexual receptivity, can shift reproductive control, and thus power, toward females (13, 22). Conversely, if males have evolved competitive traits, such as sexual dimorphism in body or canine size in the context of male–male contest competition, they can use these traits to win intersexual contests and dominate females socially, but also to coerce and monopolize females sexually, thereby preventing them from using reproductive control as a source of leverage (22). Female-biased dominance is accordingly expected to occur in species where substantial female reproductive control is manifested by i) nonpolygynous mating systems, ii) balanced adult sex ratios, iii) moderate male reproductive skew, or iv) relatively large testes (22). Additional traits that directly promote female resistance or escapes to male sexual monopolization, such as v) weak sexual dimorphism in body and canine size, vi) arboreality, vii) short sexual receptivity, and viii) high reproductive synchrony may also contribute to female biased-dominance (22).

The “female competition hypothesis” proposes that female dominance over males is a by-product of adaptations to intense reproductive or ecological female–female competition (3, 8) that selects females to invest more in intersexual contests, as often occurs in cooperatively breeding species (10, 29) or in unpredictable environments (30). This hypothesis predicts that female-biased dominance has evolved in taxa where the intensity of competition among females is manifested by i) female intolerance of other breeding females (31), ii) active regulation of the number of females in groups by means of evictions or reproductive suppression (31), and iii) reduced sexual dimorphism that is sometimes accompanied by female morphological or physiological masculinization (10). Additional traits that directly promote feeding competition, such as ecologically iv) harsh, v) variable, and vi) unpredictable environments, may also contribute to female-biased dominance (30).

The “offspring safety hypothesis” proposes that sex biases in social dominance are shaped by asymmetries in reproductive costs among the sexes, and more specifically by the risks for mothers of losing a dependent offspring in the course of an intersexual contest, by accident or due to sexually selected infanticide (32–34). Accordingly, females accompanied by vulnerable, dependent offspring are expected to actively avoid contests that pose a risk for offspring survival, especially intersexual contests in species where males are physically stronger and may be infanticidal (35–39). Where contests are unavoidable, females readily submit, as it is more costly to lose an offspring than a contest. Female-biased dominance is here expected where females have (more) opportunities to engage in intersexual contests without risks for their dependent young, namely in species where i) lactation periods are shorter relative to the interbirth interval (40), ii) mothers park offspring in nests or trees instead of carrying them permanently (41, 42), iii) males are not expected or reported to be infanticidal (43), and iv) other male or female members of the group provide regular allomaternal care decreasing the time mothers spend with offspring (44).

The remaining two hypotheses propose that sex biases in social dominance reflect the social dynamics of bisexual groups. The “female bonding hypothesis” posits that variation in female bonding and social support can shape patterns of intersexual dominance and may promote greater female dominance when females enjoy more social support than males or form coalitions to dominate males (7, 12, 45, 46). Under this hypothesis, female-biased dominance is expected in species where related females live together and regularly support each other, as i) in female-philopatric societies and ii) in groups where females exhibit high average relatedness, or iii) in species where they are known to form coalitions.

Finally, the “self-organization hypothesis” posits that variation in intersexual dominance mainly reflects variation in average adult sex ratios, which cause predictable changes in the hierarchy due to winner-loser effects (14, 47, 48). Under this last hypothesis, female-biased dominance is expected to increase as i) adult sex ratios become more male biased due to ii) a large number of males in a group, because iii) male–male fights become more frequent and generate loser males that drop to the bottom of the intersexual hierarchy, resulting in an increase of average female ranks as a by-product.

## Results

### Distribution of Sex-Biases in the Frequency and Outcome of Male–Female Contests.

**Fig. 1.**
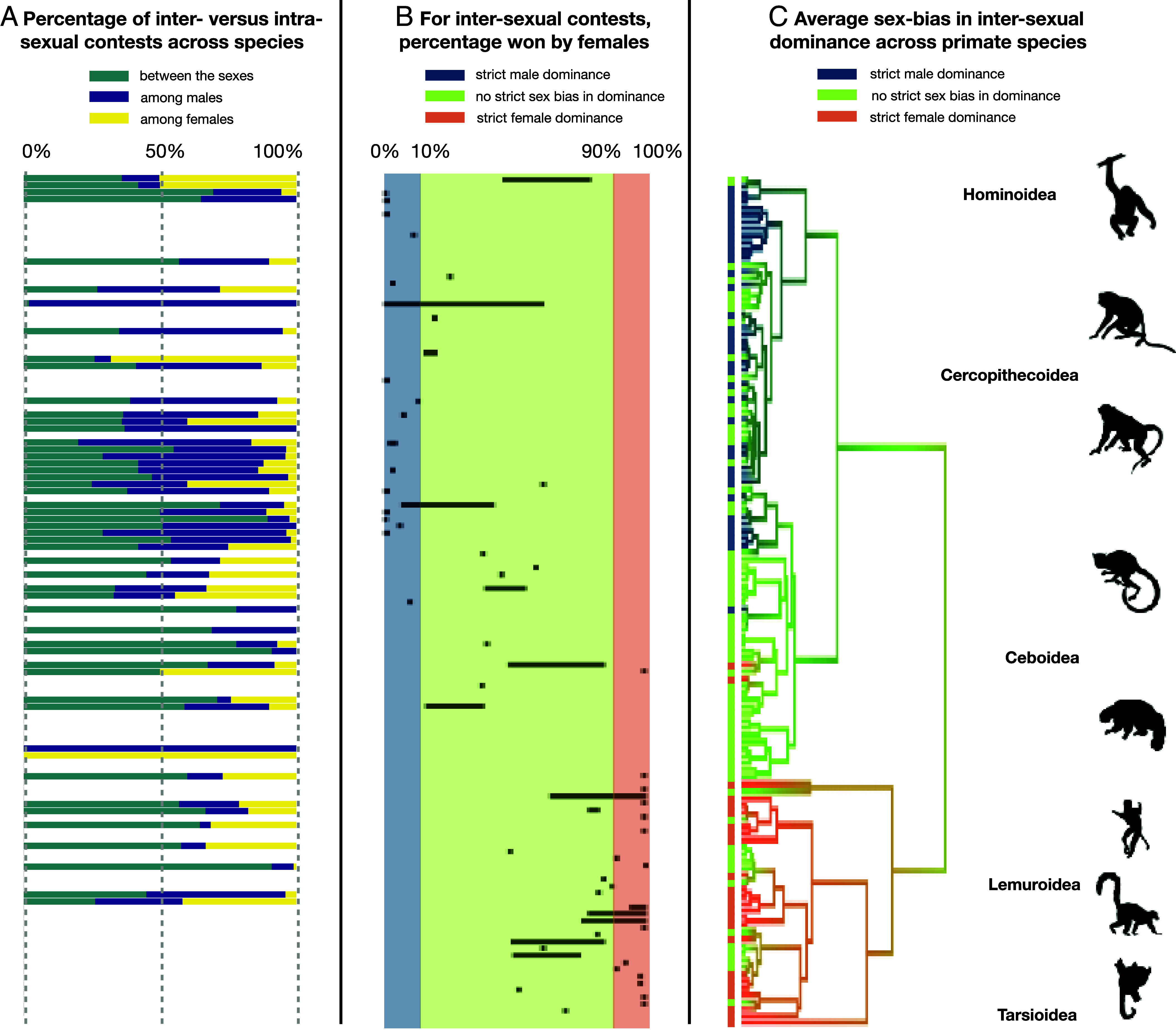
The prevalence of intersexual contests and the distribution of intersexual dominance across primate societies. (*A*) Percentage of intersexual, male–male and female–female contests for each species included in this study, ordered alongside the primate phylogeny (see Panel *C*); (*B*) observed percentage of contests won by females for each species; black horizontal bars cover the range of values observed across different populations or studies of the same species; the color code shows the link between the percentage of contests won by females and the 3-level qualitative measure of intersexual dominance used in this study; (*C*) taxonomic distribution of our 3-level qualitative measure of intersexual dominance mapped onto the most likely primate phylogeny (credit for icons: https://www.phylopic.org).

Our comprehensive review of the distribution of the frequency and outcome of male–female agonistic interactions (“contests”) yielded three insights. First, contests between males and females were surprisingly frequent, representing nearly half of all events (mean ±SD: 47.4 ± 21.9%, Fig. 1*A*), underscoring the importance of understanding their causes and consequences. The proportion of male–female aggression was not significantly correlated with the proportion of opposite-sex dyads in a group (estimate: −0.01, 89% CI: −0.06; +0.04), indicating that male–female contests are influenced by specific factors that go beyond general levels of within-group competition.

Second, male dominance over females was far from ubiquitous, and intersexual dominance varied along a continuum (Fig. 1 *B* and *C*). Therefore, we investigated sex biases in the outcome of intersexual contests with two measures (Fig. 1*B*): i) the percentage of intersexual contests won by females, and ii) a three-level qualitative variable, distinguishing between strictly female-dominant, strictly male-dominant (> 90% of intersexual contests won by one sex, or one sex reported to be “always dominant”), and moderate sex biases (≤ 90 % of intersexual contests won by one sex, meaning that both males and females can win intersexual contests, at least occasionally). Of the 151 primate populations from 84 species for which quantitative measures of intersexual contests were available (Fig. 1*B*), females virtually always won in 20 populations (13%, n = 16 species) and males in 25 populations (17%, n = 16 species), leaving 106 populations (70%, n = 69 species) with moderate sex biases.

Third, sex biases in intersexual dominance can also vary within species. Among the 52 species represented by more than one study population (Fig. 1*B*), the percentage of intersexual contests won by females exhibited extensive intraspecific variation, spanning, for example, 0 to 61% in patas monkeys *(Erythrocebus patas),* or 48 to 79% in bonobos *(Pan paniscus*). In one species (*Miopithecus talapoin*) all three patterns of intersexual dominance (strict male dominance, strict female dominance, and no strict bias in intersexual dominance) have been described for different groups. Notably, the absence of a strict sex bias in dominance may arise either because contests between males and females are rare (n = 21 populations, mostly in the *Pitheciidae*) or because there is no detectable bias in the outcome of intersexual contests, meaning that males and females are equally likely to win (n = 85 populations distributed across lineages).

Closer inspection of the taxonomic distribution of sex biases in intersexual dominance across species confirmed and quantified a previously reported pattern (5, 13) (Fig. 1*C*): Strict male dominance is mainly found among great apes and catarrhines (i.e., African and Asian monkeys), strict female dominance mainly occurs in strepsirrhines (i.e., lorises, galagos, and Malagasy lemurs), and limited or no sex bias in dominance characterizes most platyrrhines (i.e. South-American monkeys). In support of this three-branch structure, our measures of sex biases in dominance exhibited a significant phylogenetic signal (continuous variable: K = 0.38, *P* = 0.001, categorical variable: Blomberg’s K = 0.18, *P* = 0.001), indicating that more closely related species exhibit a more similar degree of sex-biased dominance. This pattern, with few evolutionary transitions from strict female dominance to strict male dominance (and *vice-versa*) limits formal ancestral state reconstructions, and suggests that such transitions are gradual rather than showing dramatic shifts (Fig. 1*C*).

### Support for Hypotheses Explaining Female-Biased Dominance.

**Fig. 2.**
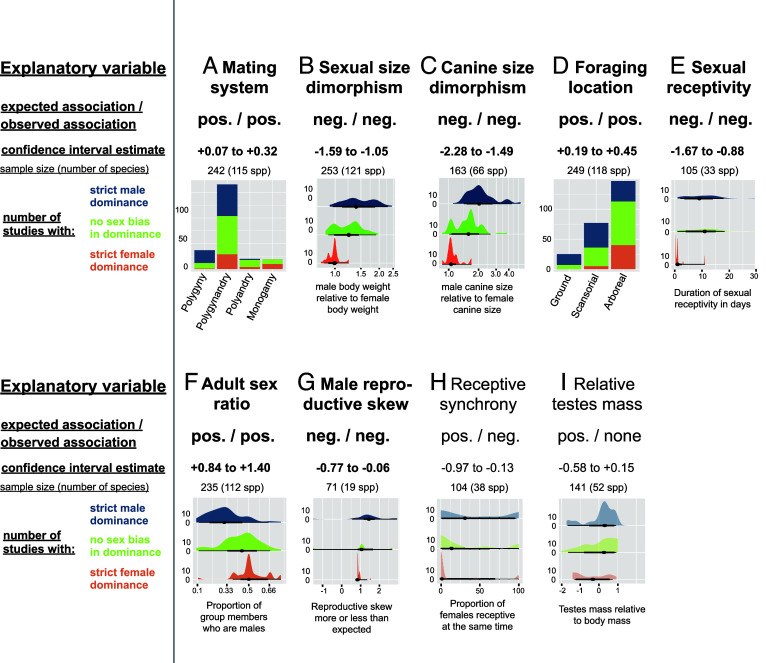
Intersexual dominance is associated with sex biases in reproductive control. Summary of phylogenetically controlled test of the “reproductive control hypothesis”, including one panel per explanatory variable: (*A*) mating system, (*B*) sexual size dimorphism, (*C*) canine size dimorphism, (*D*) foraging location, (*E*) sexual receptivity, (*F*) adult sex ratio, (*G*) male reproductive skew, (*H*) receptive synchrony, and (*I*) relative testes mass. Each panel indicates the direction of the expected and observed observation, the CI of the estimate from the Bayesian posterior (53), the sample size, and the graphical distribution of the raw observations according to whether the population is classified as having strict male dominance, no strict sex bias in dominance, or strict female dominance. Plots are shaded when the observed relationship is not in the predicted direction. See *SI Appendix,* Table S1 for full statistical results.

Our analyses revealed clear support for the “reproductive control hypothesis”(see Fig. 2 for illustration, statistical results, and sample sizes for the analyses with the categorical outcome variable classifying species as having strict female, no sex bias, or strict male dominance; for full results see *SI Appendix,* Table S1). This pattern was generally robust to the exclusion of lemurs from the main dataset (*SI Appendix,* Table S2), suggesting that the reported associations are not exclusively driven by contrasts between lemurs and other primate taxa. First, strict female dominance was particularly common in mating systems where females retain substantial reproductive control, i.e., in monogamous (53%), polyandrous (20%), and polygynandrous species (17%), compared to polygynous species (0%) (CI for comparisons between polygyny and the other three mating systems do not cross zero; Fig. 2*A*). Notably, we did not observe any case of strict male dominance in monogamous species. Second, dominance is more female biased in arboreal species where females have better chances to escape male monopolization than in terrestrial species, and in species where shorter sexual receptivity reduces the time period over which females need to resist male mating attempts (Fig. 2 *D* and *E*). Third, female-biased dominance was associated with more balanced physical power between the sexes and more even adult sex ratios (Fig. 2 *B*, *C*, and *F*). Strikingly, in primate species with no sexual dimorphism in body or canine size, females more often dominate males (median of 84% of contests won by females in monomorphic species). Moderate male-biased dimorphism can be associated with either male-biased or female-biased dominance, but dominance biases rapidly shift toward males when males are >50% heavier than females (Fig. 2 *B* and *C*), showing that only large asymmetries in physical power or weaponry between the sexes override other influences on intersexual dominance. Against our expectations, lower female reproductive synchrony, which often facilitates male monopolization of fertile females (49), was associated with greater female-biased dominance (Fig. 2*H*). This confirms findings from a recent study (13), according to which the leverage obtained by fertile females is reinforced by their scarcity via biological market effects (50). Alternatively, and perhaps more realistically, the relationship between reproductive synchrony and intersexual dominance is complicated by confounding effects, such as the length of sexual receptivity and the number of females in a group (*SI Appendix,* Tables S3 and S4). Finally, the existence and direction of associations linking intersexual dominance to relative testis size and to male reproductive skew, two imperfect proxies of male ability to monopolize females sexually (51, 52), were inconsistent across models and datasets (Fig. 2 *G* and *I* and *SI Appendix,* Tables S1 and S2).

**Fig. 3.**
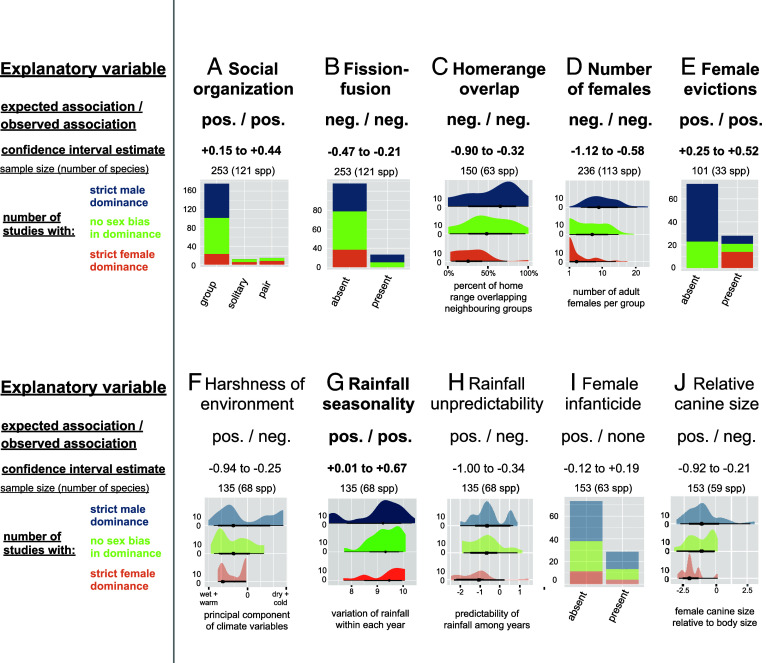
Female-biased dominance is associated with intense female competition. Summary of phylogenetically controlled test of the “female competition hypothesis”, including one panel per explanatory variable: (*A*) social organization, (*B*) fission-fusion, (*C*) home-range overlap, (*D*) number of females, (*E*) female evictions, (*F*) harshness of environment, (*G*) rainfall seasonality, (*H*) rainfall unpredictability, (*I*) female infanticide, and (*J*) relative canine size. Each panel indicates the direction of the expected and observed observation, the CI of the estimate from the Bayesian posterior (53), the sample size, and the graphical distribution of the raw observations according to whether the population is classified as having strict male dominance, no strict sex bias in dominance, or strict female dominance. Plots are shaded when the observed relationship is not in the predicted direction. See *SI Appendix,* Table S3 for full statistical results.

We also found substantial support for the “female competition hypothesis”, both in the main dataset (see Fig. 3 for illustration, main statistical results, and sample sizes; see *SI Appendix,* Table S3 for full results) and after excluding lemurs (*SI Appendix,* Table S4). First, female-biased dominance was more common, and strict male dominance was absent, in pair-living and solitary species, where females are typically intolerant of other breeding females, than in group-living species, with females winning an average of 92%, 82%, and 18% of intersexual contests in pair-living, solitary, and group-living species, respectively (Fig. 3*A*). Second, among group-living species, female-biased dominance was more common in stable groups, where females routinely compete over resources, than in species with a fission-fusion social organization, where food competition is mitigated by flexible association patterns (Fig. 3*B*). Third, females were more likely to dominate males in species where females defend more exclusive home ranges (Fig. 3*C*), as well as in groups where the number of adult females is low (Fig. 3*D*) and actively regulated by forcible evictions (Fig. 3*E*), which are all indicators of high levels of within-group female competition. Fourth, the previously reported association between female-biased dominance and sexual monomorphism in body and canine size (Fig. 2 *B* and *C*) also supports the “female competition hypothesis”, because monomorphism often reflects roughly similar levels of intrasexual competition in males and females. Nonetheless, several predictions of the “female competition hypothesis” were poorly supported, narrowing the conditions under which competition among females influences male–female dominance relations (Fig. 3 *F*–*J*). In particular, female-biased dominance was not clearly nor consistently associated with ecological factors thought to generate permanent or temporal food limitation such as i) environmental harshness characterized by dry and cold climates (Fig. 3*F*), ii) rainfall seasonality (Fig. 3*G*), and iii) rainfall unpredictability (Fig. 3*H*). In addition, we found no clear link between intersexual dominance and infanticide by females (Fig. 3*I*), which is rare in primates and restricted to specific contexts (54). Finally, and unexpectedly, females have smaller canines for their body size in species with female-biased dominance (Fig. 3*J*), possibly reflecting the distinct evolutionary trajectories of relative canine size in lemurs versus anthropoid primates (55, 56). Alternatively, this pattern may also reflect the fact that females compete via direct physical contests less often than males (22).

**Fig. 4.**
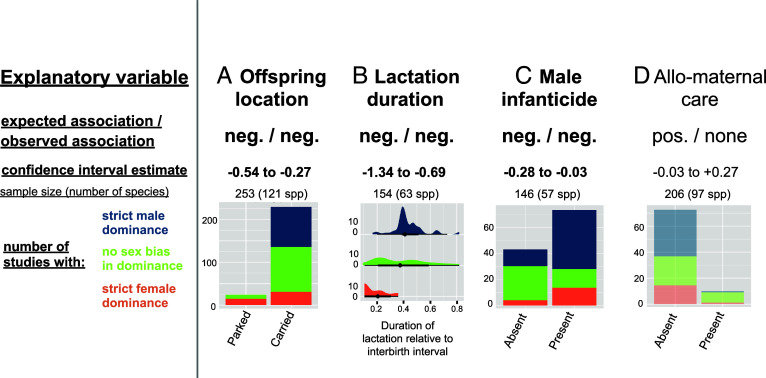
Intersexual dominance is associated with sex biases in reproductive costs. Summary of phylogenetically controlled test of the “offspring safety hypothesis”, including one panel per explanatory variable: (*A*) offspring location, (*B*) lactation duration, (*C*) male infanticide, (*D*) allo-maternal care. Each panel indicates the direction of the expected and observed observation, the CI of the estimate from the Bayesian posterior (53), the sample size, and the graphical distribution of the raw observations according to whether the population is classified as having strict male dominance, no strict sex bias in dominance, or strict female dominance. Plots are shaded when the observed relationship is not in the predicted direction. See *SI Appendix,* Table S5 for full statistical results.

Support for the “offspring safety hypothesis” was more partial (see Fig. 4 for illustration, main statistical results, and sample sizes), with patterns that differed differing across the main branches of the order Primates (*SI Appendix,* Table S5). Across all primates, female-biased dominance was, as expected, more common in species where females park infants while foraging instead of carrying them on their body (Fig. 4*A*), wean them rapidly (Fig. 4*B*) and where males have not been reported to commit infanticide (Fig. 4*C*, though the latter association only reached significance with the categorical variable of intersexual dominance). The effect of relative lactation duration disappeared in the subset excluding lemurs (*SI Appendix,* Table S5), while the association predicted between female-biased dominance and allomaternal care was only detectable in this subset, possibly because allomaternal care is relatively rare in lemurs (57).

**Fig. 5.**
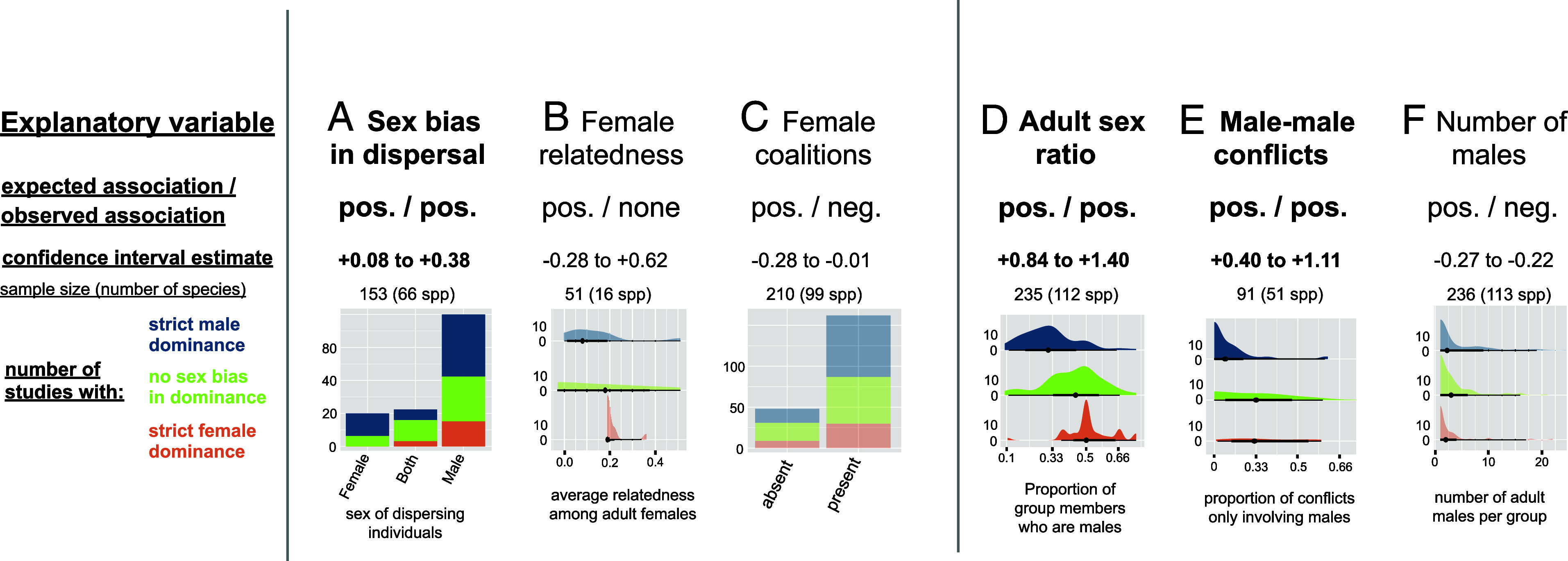
Intersexual dominance is poorly predicted by female bonding patterns or by self-organization processes structuring dominance hierarchies. Summary of phylogenetically controlled test of the test of the “female bonding” and “self-organization” hypotheses, including one panel per explanatory variable: (*A*) sex bias in dispersal, (*B*) female relatedness, (*C*) female coalitions, (*D*) adult sex ratio, (*E*) male–male conflicts, (*F*) number of males. Each panel indicates the direction of the expected and observed observation, the CI of the estimate from the Bayesian posterior (53), the sample size, and the graphical distribution of the raw observations according to whether the population is classified as having strict male dominance, no strict sex bias in dominance, or strict female dominance. Plots are shaded when the observed relationship is not in the predicted direction. See *SI Appendix,* Table S7 for full statistical results.

Support for the “female bonding hypothesis” was limited, both in the main sample (see Fig. 5 *A*–*C* for illustration, main statistical results, and sample sizes; see *SI Appendix,* Table S6 for full results), and after excluding species with large sexual size dimorphism from the sample, which might have overridden other factors (*SI Appendix,* Table S6). As expected, female-biased dominance was more common among species with female philopatry than where females disperse. However, it was neither more common in species that form groups with higher average female relatedness, nor in species where females form agonistic coalitions. The observed association between female philopatry and dominance is thus unlikely mediated by the formation of coalitions between natal, related females against immigrant males. Instead, it may reflect the fact that females can choose to stay in female-dominant societies, while they may often be forced to disperse in male-dominant societies, as in some polygynous primates, where females move between one-male units (58), and in groups where young females face the risk of mating with their father due to his long alpha tenure (59).

Finally, support for “the self-organization hypothesis”, according to which variation in intersexual dominance is context-dependent and reflects variation in adult sex ratios (14), was relatively weak (see Fig. 5 *D*–*F* for illustration, main statistical results, and sample sizes), even after excluding species with large sexual size dimorphism from the main dataset (*SI Appendix,* Table S7). As predicted, intersexual dominance shifted toward females as the adult sex ratio becomes more male biased. However, it was neither consistently associated with the proportion of male–male contests nor with the number of males in the group (*SI Appendix,* Table S7), refuting two core predictions of this hypothesis.

## Discussion

By quantifying male–female dominance relationships across primates and exploring key evolutionary scenarios that may have shaped their variation, our study considerably enhances our understanding of their distribution and evolution. We found substantial variation in intersexual dominance relationships within and between species, emphasizing that in most primate populations both females and males can win contests against individuals of the opposite sex. We tested five hypotheses to explain the observed variation in sex-biased dominance, all of which rely on general mechanisms that are applicable beyond primates. Our results primarily support two hypotheses, namely that i) when one sex exerts clear reproductive control it also socially dominates the other, and ii) females have a competitive advantage over males in species where female–female competition is intense. We also found some support for a competitive disadvantage of females in species where they face intense reproductive costs.

Our results revealed multiple robust associations between intersexual dominance and a suite of social, morphological, and life-history traits. Our findings expand beyond previous studies, which focused on the links of intersexual dominance with sexual dimorphism in body size and canine size as well as with group sex ratio (3, 13). Here, we show that female-biased dominance is most often found in primate societies where individuals live alone or in pairs (by contrast to group-living) and exhibit monogamous, polyandrous, or polygynandrous mating systems (by contrast to polygyny). Although relatively rare in group-living species, female-biased dominance occurs most commonly in stable groups (by contrast to fission-fusion dynamics) with balanced adult sex ratios, where the number of adult females per group is low and actively regulated by aggressive evictions. Life-history traits associated with female-biased dominance include sexual monomorphism in body and canine size, arboreality, low receptive synchrony and short sexual receptivity in females, and female philopatry. With less certainty, populations exhibiting female-biased dominance tend to be associated with shorter lactation periods, mothers who park instead of carry infants while foraging, the presence of allomaternal care and no male infanticide. Male-biased dominance was instead most commonly observed in group-living species with polygynous or polygynandrous mating systems, fission-fusion dynamics, female-biased adult sex ratios, and a larger number of adult females in groups. It was further found to be associated with male-biased dimorphism in body and canine size, terrestriality, and female dispersal. Overall, portraying the theoretical species ranging at each end of the intersexual dominance continuum—from strict female dominance to strict male dominance—opens avenues for identifying the conditions of emergence of female- (versus male-) biased dominance in primate evolutionary history.

Some traits facilitating female reproductive control, such as sexual monomorphism, arboreality, or short sexual receptivity were associated with female-biased dominance (22). Multiple other morphological, physiological, or behavioral traits allow females to gain more control over reproduction (22), but their association with female-biased dominance is difficult to test using comparative approaches because females from different taxa use distinct strategies. For example, female bonobos have extended sexual receptivity using unreliable fertility signals that prevent male monopolization during ovulation (60), while female lemurs exhibit very short sexual receptivity, which facilitates their resistance to monopolization (61). Conversely, our study illuminates the evolutionary pathways linking male reproductive control to male-biased dominance. In particular, our results point to the evolution of terrestriality as a decisive milestone in the history of male–female power relationships in primates. By fostering male abilities to defend more females while limiting female possibilities to escape, greater terrestriality has shifted reproductive control toward males, promoting polygyny, male–male contests over females, and hence larger male bodies or weapons, thereby enhancing male potential for sexual and social coercion (reviewed by refs. 22 and 52). Our analyses support key steps of this scenario (Fig. 2), by showing associations between male-biased dominance and terrestriality, polygyny, female-biased adult sex ratios in groups, and male-biased sexual size and canine dimorphism. The generality of this scenario could be tested in other mammalian groups, such as pinnipeds, where matings can occur on land versus at sea, with important consequences for male monopolization potential and mating systems (62).

Our results further indicate that female-biased dominance covaries with multiple morphological and behavioral indices of female–female competition, but such competitive environments are poorly predicted by climatic variables. This pattern does not support hypotheses positing that female-biased dominance in lemurs evolved as an adaptive response to the harsh and variable environments of Madagascar (30, 63). However, this interpretation should be tempered by the limitations of satellite imaging in accurately capturing fine-scale variation in primate food availability (64). Moreover, the larger reproductive costs faced by females (compared to males) likely widen power asymmetries between the sexes, which may partly explain why male-biased dominance is more common in catarrhines than in strepsirrhines. As mother–infant associations lengthen and strengthen, due to slower development and more protective and exclusive maternal strategies, females may increasingly refrain from contests with males because of the associated risks of injuries for their dependent infants.

While female-biased dominance was more frequent in species with female philopatry, it was not associated with the occurrence of female–female coalitions, suggesting that sex-biased dispersal patterns may be a consequence, rather than a cause of sex biases in dominance. Similarly, in humans, gender biases in residency are apparently absent or limited in mobile hunter-gatherers due to the lack of gender inequalities and could have emerged during transitions to agriculture due to growing inequalities (65). Nevertheless, the lack of association between female coalitions and female-biased dominance fails to support a hypothesis originally proposed to explain the contrast in intersexual dominance between bonobos and chimpanzees (12, 46). The intense bonds and frequent coalitions of female bonobos may represent an unusual or partial pathway to individual female-biased dominance (46). Coalitions among females may still contribute to shift power toward females in synergy with other mechanisms, as in spotted hyenas, where females have full reproductive control (66) and rely on social support to dominate males (22, 67). Finally, the observed associations between intersexual dominance, adult sex ratio, and number of males in a group did not follow the dynamics predicted by *the self-organization hypothesis*, and may instead reflect variation in social organization and mating systems across species. Nevertheless, *the self-organization hypothesis* is better tested using modeling and population-level studies (14, 47, 48), and may still be valid under particular conditions (23).

This study should be regarded as an important step, rather than the last word, for our understanding of the evolution of intersexual dominance relations. Our primary objective was to match published, quantitative data on intersexual contests with a testable framework enabling us to identify the most promising hypotheses among those that were previously proposed. This framework guided our analytical approach based on targeted, univariate correlations across primates. Nevertheless, some of the explanatory factors we assessed are not independent of each other, calling for more detailed, multivariate tests of those hypotheses that were supported by our findings. It is also likely that there are different, conditional pathways that explain whether females or males dominate the opposite sex in a particular lineage. Finally, these hypotheses may often operate in synergy. For example, intense female reproductive competition can, by reducing power asymmetries between males and females, promote mechanisms of power acquisition that are not based on physical superiority, such as leverage effects favoring female reproductive control (13, 30, 68). Taken together, these results could guide future studies toward priority predictions. For example, the occurrence of female-biased dominance in cooperatively breeding mammals (10, 11) may be explained by the intense reproductive competition that females face for allomaternal care, alongside the alleviation of their maternal costs. Studies in other mammalian orders will allow future assessments of the generality of these conclusions, when more data on intersexual dominance become available.

Finally, our study may also contribute to a better understanding of the evolutionary origins of power biases in humans. Our comparative approach can help to identify human specifics by placing them along the spectrum of intersexual power observed in nonhuman societies. We show that our primate relatives exhibit every possible pattern from strict female to strict male power and that sex biases are absent to moderate in most primate populations. Given that humans are moderately sexually dimorphic and exhibit unusually flexible social organizations and mating systems, intersexual power in our ancestors was likely moderately sex biased (or unbiased), as in some of our closest relatives (12, 46). By tracing the evolutionary history of male–female dominance relations in our primate relatives, our study makes a decisive step toward disentangling the relative roles played by our evolutionary legacy, ecology, and cultural norms and institutions in shaping human gender biases in power.

## Materials and Methods

### Data Collection.

We combined information from literature searches with records from existing comparative datasets to assess whether differences in intersexual dominance across populations and species are associated with a set of predictor variables. We provide the data collected specifically for this project with the respective references in *SI Appendix,* Table S8. The full dataset is available at Edmond https://doi.org/10.17617/3.OOC1BX (69).

### Data on Intersexual Dominance.

We conducted a nonsystematic literature search in the *Web of Science* and *Google Scholar* databases using the key word combinations: (dominance* OR hierarchy* OR aggression* OR conflict* OR agonism* OR intersexual*) AND (primates* OR the respective primate family* OR the respective primate species*). We searched for information for each primate species, following the taxonomy of Burgin et al. (70). The literature search was conducted between May 2020 and July 2024. For all searches, we checked the titles and abstracts of the first sets of articles as automatically sorted by the respective search engine to identify studies that reported on aggressive interactions between the sexes in the respective species. From the relevant papers, we extracted information on intersexual dominance. Where available, we directly recorded the number of dyadic intersexual contests won by adult females and by adult males and the total number of contests. Contests included both physical altercations and signals in both aggressive and submissive contexts, and the information needed to clearly indicate which individual won and which lost the interaction (as for example displayed in hierarchy matrices). From some articles, we could only extract summarized information on the percentage of intersexual interactions won by either sex, and from some others, only qualitative information on whether individuals of one sex are consistently dominant over individuals of the opposite sex. We extracted this information, whenever possible, for each population, and for each social group, of a given study; otherwise, we used the mean across all groups (for quantitative variables). We only recorded contests between adults; contests involving subadults were systematically excluded. Studies that included subadults without giving the necessary details to identify and exclude the relevant subadult records were also discarded. Based on this information, we generated two variables to describe the extent of intersexual dominance: first, the percentage of intersexual contests won by females (quantitative measure), and second, a three-level qualitative variable classifying a species population as strictly female dominant, strictly male dominant, or whether neither sex is strictly dominant. A population was classified as showing strict dominance when either females or males won more than 90% of contests or when a qualitative statement clearly indicated that individuals of one sex always won intersexual contests. Interobserver reliability checks showed that our definitions for data extraction were consistent because the classification of two independent scorers was the same for 95% of a random subset of 40 studies. Each author extracted data from a subset of species, and each data point was cross-checked by a second observer. The remaining disagreements were about whether a given study had a sufficient sample to be included. We resolved all cases where there were questions about whether a study should be included or not jointly. We recorded if data came from captive or natural populations, and initial data exploration revealed no significant difference between captive and natural populations for our main response variables. For seven species, where we had data from both captive and natural populations, captivity was associated with small, inconsistent changes in the estimated degree of intersexual power. In three species, females won slightly more contests in captive than in natural populations; in one species, the proportion of contests won by females was identical in captive and natural populations; and in the remaining three species, females won slightly fewer contests in captive than in natural populations. We retained records from captive populations because discarding them would have substantially reduced the sample size.

### Data on the Frequency of Intersexual Contests.

For the subset of our sample of studies on intersexual dominance that reported the number of contests among pairs of individuals with known sex, we calculated the proportion of interactions occurring between the sexes or within either sex. We compared only sex-specific proportions because the differences in approaches used to record intersexual contests across the studies in our sample made standardization of aggression rates impossible. Data were included for groups which contained individuals of both sexes and two or more individuals of at least one sex to compare the relative occurrence of contests within versus between the sexes.

### Data on Predictor Variables.

Wherever possible, we recorded group composition in terms of numbers of adult females and adult males directly from the articles providing the data on intersexual dominance. Information for all other variables was not available on a group or population level, and we extracted information from previously published datasets describing the most likely average state within a given species instead. The variables linked to each of our five hypotheses are defined and described in the supplementary file.

### Information on Phylogenetic Relatedness.

For the primate species in our sample, we generated a consensus phylogenetic tree based on a recent complete mammalian time-calibrated phylogeny (71). We downloaded a credible set of 1,000 trees fromvertlife.org/phylosubsets/ (August 2024) and used TreeAnnotator [version 1.10 in BEAST (72)] to generate a maximum clade credibility (MCC) tree (median node heights and a burn-in of 250 trees). We include the tree as *SI Appendix*.

### Statistical Analyses.

We performed all analyses in the statistical software R version 4.2.2 (73). We provide the code at https://github.com/dieterlukas/primate_power.

### Estimation of Phylogenetic Signal.

We estimated the strength of the phylogenetic signal for both the quantitative and the qualitative measure of intersexual dominance using the function phylosig in the package “phytools” (74) in R to calculate the *K*-statistic (75), assessing their significance by comparison to 10,000 simulations. We reconstructed a likely phylogenetic history of the qualitative measure of intersexual dominance using the function contMap in the package “phytools” for plotting in Fig. 1.

### Estimation of Associations Among Variables.

We used functions of the package “rethinking” (53) to write the models to estimate associations between intersexual dominance and the respective predictor variables with Markov Chain Monte Carlo procedures in Stan (76). We built separate models for each predictor variable to estimate whether the variable had the predicted overall effect on variation in intersexual dominance. The outcome variable was the respective measure of intersexual dominance, coded first as the percentage of contests won by females; and second as ordered categorical variable with the three following categories: strict female dominance, no strict sex bias in intersexual dominance, and strict male dominance. We standardized all quantitative predictor variables by subtracting the mean from each value and then dividing by the SD and provided the same weakly regularizing prior for all.

We linked the quantitative measure of intersexual dominance, the percentage of contests won by females, to the predictor variables in a binomial model with a logit link (for details, see *SI Appendix*). We also used such a model to link the proportion of contests won by females to the proportion of dyads in each group that are between individuals of the opposite sex. We linked the three-level classification of sex bias in intersexual dominance as discrete ordered categories to the respective predictor variables using a cumulative link function, the log-cumulative-odds that a response value is equal to or less than some possible outcome (for details, see *SI Appendix*). Both types of statistical models accounted for the potential similarity among observations that might arise through shared phylogenetic history. Observations from multiple populations of the same species are nested within this phylogenetic covariance structure, assuming that these observations should have the highest likelihood of being identical. We first ran models on the main dataset, then on subsets depending on these results. When a given hypothesis was supported, we retested it after excluding lemurs, to confirm that the observed associations were not simply driven by contrasts between lemurs (where dominance is heavily female biased) and nonlemurs (where dominance is unbiased or male biased). When a given hypothesis was unsupported, we retested it after excluding the most sexually dimorphic species (i.e., where male body mass is higher than female body mass*1.10) to ensure that the associations of interest were not overridden by the effect of sexual size dimorphism.

For each model, we drew 8,000 samples from four chains, checking that for each the Gelman–Rubin convergence diagnostic “R-hat” values are less than 1.01 indicating that the Markov Chains have converged toward the final estimates. For all relationships, following the social convention introduced for Bayesian analyses (53), we report the mean and the 89% compatibility interval (CI) of the posterior of the estimated association between each predictor and the outcome variables. We assumed that a predictor variable is reliably associated with the respective measure of intersexual dominance in our data if this interval does not include zero.

In the main text (Figs. 2–5), we present the results from the analyses with the discrete outcome variable (strict female bias, no sex bias, strict male bias) because the results were generally consistent independently of which outcome variable we used (we mention the single variable for which this was not the case). The full statistical results can be found in *SI Appendix,* Tables S1–S7.

## Supplementary Material

Appendix 01 (PDF)

## Data Availability

All data and code required to repeat the analyses are available at Edmond (https://doi.org/10.17617/3.OOC1BX) (69).
