# Supplementary material for: The evolution of male–female dominance relations in primate societies
Source: Proc Natl Acad Sci U S A. 2025 Jul 7;122(28):e2500405122. doi: 10.1073/pnas.2500405122 (PMC12280975; doi:10.1073/pnas.2500405122)
Supplement: Supplementary file 1 — Appendix 01 (PDF) [file pnas.2500405122.sapp.pdf]

Supplementary information for:

The evolution of male-female dominance relations in primate societies

Authors: Elise Huchard[1]*†, Peter M. Kappeler[2,3]†, Nikolaos Smit[1], Claudia Fichtel[2], Dieter Lukas[4*]

Affiliations:

[1]ISEM, University of Montpellier, CNRS, IRD, Montpellier, France

[2]Behavioral Ecology and Sociobiology Unit, German Primate Center, Leibniz Institute for Primate Research, Göttingen, Germany

[3]Department of Sociobiology/Anthropology, Johann-Friedrich-Blumenbach Institute of Zoology and Anthropology, University of Göttingen, Göttingen, Germany

[4]Department of Human Behavior, Ecology and Culture, Max Planck Institute for Evolutionary Anthropology, Leipzig, Germany

*Co-corresponding authors. Email: elise.huchard@umontpellier.fr; dieter.lukas@gmail.com

† These authors contributed equally to this work.

Table of contents:

Supplementary methods pp 2-7

Supplementary references pp 8-10

Supplementary results: Tables S1-S7 pp 11-17

Supplementary Table S8: original data with references pp 18-55

Supplementary File 1: phylogenetic tree pp 56-58

Supplementary methods

(1) Description of variables

Reproductive control hypothesis

- **mating system**, coded as polygynous (most males mate with multiple partners and most females mate with a single partner in a given mating season), polygynandrous (most males and most females mate with multiple partners), monogamous (most males and females mate with a single partner), and polyandrous (most females mate with multiple partners and most males mate with a single female). The mating system is an indicator of which sex controls reproduction (1). Data are from (2, 3).
- **main strata of activity**, coded as whether individuals of the species primarily forage on land (ground), scansorial (both ground and arboreal), or in trees (arboreal). Terrestriality directly promotes male reproductive control while arboreality promotes female reproductive control by facilitating their escape to male monopolization attempts (1). Data are from (4).
- **sexual receptivity duration**, calculated as the number of hours females are receptive each month. Short receptivity periods promote female reproductive control by limiting the time over which females need to resist to male monopolization attempts (1, 5). Data are from (6, 7).
- **sexual dimorphism in body mass**, calculated using the two-step approach of Smith (1999) as male mass divided by female mass if males are heavier, and 2 minus female mass divided by male mass if females are heavier. Large (male-biased) sexual dimorphism in body size is both an indicator and a driver of male reproductive control, by facilitating the use of sexual and social coercion by males (1). Data are from (2, 8–12).
- **sexual dimorphism in canine size**, calculated using the two step approach of Smith (1999) as male canine length divided by female canine length if males have larger canines and as 2 minus female canine length divided by male canine length if females have larger canines. Larger (male-biased) dimorphism in canine size is both an indicator and a driver of which sex controls reproduction, reflecting the intensity of male-male contest competition (typically linked to male ability to monopolize multiple females) and facilitating the use of sexual and social coercion by males (1). Data are from (3, 13).

- **adult sex ratio in the social group**, following (14), calculated as the number of adult males in the group divided by the sum of the numbers of adult males and females in the group. Female-biased adult sex ratio is an indicator of male reproductive control, reflecting their ability to defend groups of females (1). Data from the original studies.
- **skew in reproductive success among multiple males in the social group**, calculated using the M-index as deviation from the expected share if all males randomly gain mating success or paternity. Larger male reproductive skew is an indicator of male reproductive control and monopolization potential (1). Data are from (15).
- **relative testes size**, calculated as the species-level residual of a correlation between average testes weight and average body weight. Larger relative testes size is an indicator of female reproductive control, reflecting their capacity to evade male monopolization and mate promiscuously (1). Data are from (16).
- **receptive synchrony**, calculated as the probability that two or more females in a social group are receptive/mating on the same day. Larger receptive synchrony promotes female reproductive control, by facilitating female ability to evade male monopolization (1). Data are from (17, 18).

Female competition hypothesis

- **social organization**, coded as solitary (general activity of individuals is not synchronized across extended time spans), pair living (permanent association of one adult female and one adult male), and group living (stable association of at least two same-sex individuals plus potential opposite sex individuals). Social organization is an indicator of female-female competition, reflecting female tolerance of other females (19). Data are from (20).
- **home range overlap**, calculated as the percentage of overlap between the home ranges of neighboring solitary individuals/pairs/groups. Home range overlap is an indicator of female-female competition, reflecting their tolerance to the spatial proximity of other females (21). Data are from (22).
- **presence of a fission-fusion social system,** coded as whether female group members form flexible subunits as a social adaptation to mitigate feeding competition (23), leading to fluctuating patterns in group cohesion or whether they live in stable groups, where they cannot avoid feeding competition. Data are from (24, 25).

- **number of adult females in the social group**, where low numbers of females usually reflect limitations in group size imposed by intense resource competition between females (19). Data from the original studies.
- **presence of female evictions**, coded as whether or not females forcibly evict other subordinate females from their social groups, which is a direct manifestation of the intensity of resource or reproductive competition among females (19). Data are from (26).
- **presence of female infanticide**, coded as whether or not females kill dependent offspring of other females, which is direct manifestation of the intensity of reproductive competition among females (19). Data are from (26).
- **female canine size,** the length of the upper canines of females in centimeters; and female body mass in grams, to derive relative canine sizes. Relative female canine size is an indicator of the intensity of female-female contest competition (19). Data are from (3, 13).
- **environmental harshness** across the range of a species, calculated as the principal component of a multivariate analysis combining temperature and rainfall information with colder and drier climates having higher harshness scores, which may increase the intensity of resource competition among females (27). Data are from (28).
- **rainfall seasonality,** calculated as the variance in annual precipitation across the range of a species. A higher seasonality is generally expected to increase the intensity of resource competition among females during the lean season. Data are from (28).
- **predictability of annual rainfall,** calculated via Colwell's P, an information-theory-based index that captures variation in the onset, intensity and duration of annual rainfall patterns, ranging from 0 (completely unpredictable) to 1 (fully predictable). A less predictable environment is generally expected to increase the intensity of resource competition among females during dry periods. Data are from (28).

Offspring safety hypothesis

- **duration of lactation relative to interbirth interval**, calculated as the average length of lactation (in days) divided by the average length of an interbirth interval, reflecting the fraction of the maternal reproductive career during which offspring are at risk. Data are from (29).

- **mother-offspring permanent association**, coded as a binary variable reflecting whether females leave or park their offspring, vs. carry them while foraging. Data are from (30–32).
- **infanticide by males**, coded as a binary variable reflecting whether males have been reported to engage in infant killing regularly or occasionally, versus have never been reported to kill infants in a given species. Data are from (16).
- **presence of allomaternal care**, coded as a binary variable reflecting whether male or female members of a social group engage in allomaternal care, including infant carrying. Data are from (21, 33, 34).

Female bonds hypothesis

- **sex bias in dispersal**, coded as one of three categories whether most females, most males, or most individuals of both sexes move to a new group/location to start breeding, reflecting whether females have kin available for social support. Data are from (35).
- **average levels of kinship among adult females in the social group**, calculated based on kinship among all pairs of individuals in a social group derived either from pedigrees (r~0 unrelated, r=0.5 mother-daughter/full-siblings) or from levels of allele sharing across genetic loci, reflecting whether females have kin available for social support. Data are from (36).
- **coalition formation among females**, coded as whether or not aggressive interactions among group females sometimes involve two or more females joining together against other females (versus the absence of such joint interactions). Data are from (36).

Self-organization hypothesis

- **adult sex ratio in the social group**: following (14), it was calculated as the number of adult males in the group divided by the sum of the numbers of adult males and females in the group, and reflects the extent to which individuals of one sex experience more contests than others. Data from the original studies.
- **number of adult males in the social group**, reflecting whether adult sex ratio varies as a result of an increase in the number of males (versus in the number of females), as proposed in the original self-organization hypothesis (37). Data from the original studies.

- **proportion of male-male contests**, from the information on the frequency of intra- and intersexual contests, reflecting whether males are more likely to experience winner-loser effects than females. Data from the original studies.

(2) Description of statistical models

<u>Model structure to associate predictor variables with the quantitative measure of intersexual dominance (percentage of fights won by females)</u>

The quantitative measure of intersexual dominance, *quant_isd*, is the percentage of fights won by females, which can range between 0 and 1. We linked this proportion to the respective predictor variables using a logit link while taking into account that values from closely related species might be more similar. We specified the model as:

quant_isd ~ Binomial (1,p)
$\text{logit}(p) = a_{species} + b * \text{predictor}$
b ~ Normal(0,1)
$a_{species} \sim \text{MVNormal}(0,S_{ij})$
$S(i,j) = \eta^2 \exp(-\rho^2 D_{ij})$
$\rho^2 = \text{Exponential}(1)$
$\eta^2 = \text{Exponential}(1)$

where *predictor* is the place holder for the respective standardized predictor variable that links to the outcome via the slope *b* for which we set a weakly regularizing prior centered on zero assuming a priori no relationship but allowing both positive and negative relationships, $a_{species}$ reflects the separate intercepts for each species that are linked through a similarity matrix S_{ij}, which takes into account the provided pairwise phylogenetic distance D among species i and j to predict the average similarity η^2 among closely related species pairs and ρ^2 reflecting how this similarity declines as species share less phylogenetic history.

<u>Model structure to associate predictor variables with the qualitative measure of intersexual dominance (classification of strict or no sex bias in dominance)</u>

We represented the three-level classification of sex bias in intersexual dominance as discrete ordered categories (cat_isd_i, where cat_isd_1 equals strict male-biased dominance, cat_isd_2

equals no strict sex bias in dominance, and cat_isd_3 equals strict female-biased dominance). The decision to treat these categories as ordered from 1 to 3 was based on the way the classification was derived from the quantitative measure and the observation that the two strictly biased dominance categories never occurred next to each other in the same species or family but always in the presence of the intermediate category. We linked these ordered categories to the respective predictor variables using a cumulative link function, the log-cumulative-odds that a response value is equal-to-or-less-than some possible outcome, and again reflected potential similarity that might arise through shared phylogenetic history. We specified the model as:

$cat\_isd_i \sim \text{Categorical}(p)$

$p_1 = q_1$, $p_2 = q_2 - q_1$, $p_3 = 1 - q_2$

$\text{logit}(q_k) = \kappa_k - phi_i$

$phi_i = a_{species} + b * predictor$

$a_{species} \sim \text{MVNormal}(0,S_{ij})$

$S(i,j) = \eta^2 \exp(-\rho^2 D_{ij})$

$\rho^2 = \text{Exponential}(1)$

$\eta^2 = \text{Exponential}(1)$

$\kappa_k \sim \text{Normal}(0,1.5)$

where p reflects the average probability of each of the three categories, κ_k is the common prior for each of the k = i – 1 = 2 intercepts, phi_i represents the linear model for the cumulative probability for each of the i (three) categories, *predictor* is the place holder for the respective standardized predictor variable that links to the outcome via the slope b for which we set a weakly regularizing prior centered on zero assuming a priori no relationship but allowing both positive and negative relationships, and $a_{species}$ reflects the separate intercepts for each species that are linked through a similarity matrix S_{ij}, which takes into account the pairwise phylogenetic distance D among species i and j to predict the average similarity η^2 among closely related species pairs and ρ^2 reflecting how this similarity declines as species share less phylogenetic history.

Supplementary references

1. E Davidian, M Surbeck, D Lukas, PM Kappeler, E Huchard, The eco-evolutionary landscape of power relationships between males and females. *Trends Ecol Evol* **37**, 706–718 (2022).
2. PM Kappeler, CL Nunn, AQ Vining, SM Goodman, Evolutionary dynamics of sexual size dimorphism in non-volant mammals following their independent colonization of Madagascar. *Sci Rep* **9**, 1–14 (2019).
3. S Lüpold, LW Simmons, CC Grueter, Sexual ornaments but not weapons trade off against testes size in primates. *Proc R Soc B* **286**, 20182542 (2019).
4. H Wilman, et al., EltonTraits 1.0: Species-level foraging attributes of the world's birds and mammals. *Ecology* **95**, 2027–2027 (2014).
5. E Huchard, et al., Convenience polyandry or convenience polygyny? Costly sex under female control in a promiscuous primate. *Proc R Soc B* **279**, 1371–1379 (2012).
6. N Kutsukake, CL Nunn, Comparative tests of reproductive skew in male primates: The roles of demographic factors and incomplete control. *Behav Ecol Sociobiol* **60**, 695–706 (2006).
7. P Stockley, Sperm competition risk and male genital anatomy: Comparative evidence for reduced duration of female sexual receptivity in primates with penile spines. *Evol Ecol* **16**, 123–137 (2002).
8. P Jarman, Mating system and sexual dimorphism in large terrestrial, mammalian herbivores. *Biol Rev* **58**, 485–520 (1983).
9. A Loison, JM Gaillard, C Pélabon, NG Yoccoz, What factors shape sexual size dimorphism in ungulates? *Evol Ecol Res* **1**, 611–633 (1999).
10. RJ Smith, JM Cheverud, Scaling of sexual dimorphism in body mass: A phylogenetic analysis of Rensch's Rule in primates. *Int J Primatol* **23**, 41 (2002).
11. JL Isaak, Potential causes and life-history consequences of sexual size dimorphism in mammals. *Mamm Rev* **35**, 101–115 (2005).
12. SA Heldstab, et al., Reproductive seasonality in primates: Patterns, concepts and unsolved questions. *Biol Rev* **96**, 66–88 (2021).
13. JM Plavcan, CP van Schaik, PM Kappeler, Competition, coalitions and canine size in primates. *J Hum Evol* **28**, 245–276 (1995).

14. S Ancona, FV Denes, O Krüger, T Székely, SR Bessinger, Estimating adult sex ratios in nature. *Philos Trans R Soc B* **372**, 20160315 (2017).
15. CT Ross, et al., The multinomial index: A robust measure of reproductive skew. *Proc R Soc B* **287**, 20202025 (2020).
16. D Lukas, E Huchard, The evolution of infanticide by males in mammalian societies. *Science* **346**, 841–844 (2014).
17. LM Carnes, CL Nunn, RJ Lewis, Effects of the distribution of female primates on the number of males. *PLoS One* **6**, e19853 (2011).
18. J Gogarten, A Koenig, Reproductive seasonality is a poor predictor of receptive synchrony and male reproductive skew among nonhuman primates. *Behav Ecol Sociobiol* **67**, 123–134 (2013).
19. T Clutton-Brock, E Huchard, Social competition and its consequences in female mammals. *J Zool* **289**, 151–171 (2013).
20. PM Kappeler, L Pozzi, Evolutionary transitions toward pair living in nonhuman primates as stepping stones toward more complex societies. *Sci Adv* **5**, eaay1276 (2019).
21. D Lukas, TH Clutton-Brock, The evolution of social monogamy in mammals. *Science* **341**, 526–530 (2013).
22. F Pearce, C Carbone, G Cowlishaw, NJB Isaac, Space-use scaling and home range overlap in primates. *Proc R Soc B* **280**, 20122122 (2013).
23. AE Pusey, K Schroepfer-Walker, Female competition in chimpanzees. *Philos Trans R Soc B* **368**, 20130077 (2013).
24. CC Grueter, B Chapais, D Zinner, Evolution of multilevel social systems in nonhuman primates and humans. *Int J Primatol* **33**, 1002–1037 (2012).
25. KB Strier, PC Lee, AR Ives, Behavioral flexibility and the evolution of primate social states. *PLoS One* **9**, e114099 (2014).
26. D Lukas, E Huchard, The evolution of infanticide by females in mammals. *Philos Trans R Soc B* **374**, 20180075 (2019).
27. D Lukas, T Clutton-Brock, Climate and the distribution of cooperative breeding in mammals. *R Soc Open Sci* **4**, 160897 (2017).
28. CA Botero, R Dor, CM McCain, RJ Safran, Environmental harshness is positively correlated with intraspecific divergence in mammals and birds. *Mol Ecol* **23**, 259–268 (2014).
29. JP de Magalhães, J Costa, A database of vertebrate longevity records and their relation to other life-history traits. *J Evol Biol* **22**, 1770–1774 (2009).

30. PM Kappeler, Nests, tree holes, and the evolution of primate life histories. *Am J Primatol* **46**, 7–33 (1998).
31. C Ross, Park or ride? Evolution of infant carrying in primates. *Int J Primatol* **22**, 749–771 (2001).
32. SR Tecot, AL Baden, NK Romine, JM Kamilar, Infant parking and nesting, not allomaternal care, influence Malagasy primate life histories. *Behav Ecol Sociobiol* **66**, 1375–1386 (2012).
33. D Lukas, T Clutton-Brock, Cooperative breeding and monogamy in mammalian societies. *Proc R Soc B* **279**, 2151–2156 (2012).
34. K Isler, CP van Schaik, Allomaternal care, life history and brain size evolution in mammals. *J Hum Evol* **63**, 52–63 (2012).
35. T Barsbai, D Lukas, A Pondorfer, Local convergence of behavior across species. *Science* **371**, 292–295 (2021).
36. D Lukas, TH Clutton-Brock, Social complexity and kinship in animal societies. *Ecol Lett* **21**, 1129–1134 (2018).
37. CK Hemelrijk, Self-organizing properties of primate social behavior: A hypothesis for intersexual rank overlap in chimpanzees and bonobos. *Evol Anthropol* **11**, 91–94 (2003).

Table S1. Summary of the phylogenetically-controlled tests of the predictions of the 'reproductive control' hypothesis. The direction of expected (Exp.) and observed (Obs.) associations is compared to assess support for each prediction; results in support of the predictions are highlighted in bold. The confidence interval of significant associations does not include 0.

Explanatory variables		Exp. / Obs.	CI Estimate		N. obs. (N. sp.)
			Lower	Upper	
(1) Categorical response variable (strict male dominance, no strict bias in dominance, strict female dominance), full sample					
**Mating system**	**Monogamy vs. polygyny**	**pos. / pos.**	**0.280**	**0.568**	242 (115)
	**Monogamy vs. polygynandry**	**pos. / pos.**	**0.100**	**0.376**	
	**Monogamy vs. polyandry**	**none / none**	**-0.017**	**0.339**	
	**Polygynandry vs. polygyny**	**pos. / pos.**	**0.068**	**0.315**	
	**Polyandry vs. polygyny**	**pos. / pos.**	**0.092**	**0.435**	
	Polyandry vs. **polygynandry**	pos. / none	-0.091	0.238	
**Substrate use**	**Arboreal (vs. terrestrial)**	**pos. / pos.**	**0.239**	**0.520**	249 (118)
	**Arboreal (vs. scansorial)**	**pos. / pos.**	**0.185**	**0.445**	
	Terrestrial (vs. scansorial)	neg. / none	-0.230	0.106	
**Length of sexual receptivity**		**neg. / neg.**	**-1.664**	**-0.883**	105 (33)
**Sexual size dimorphism**		**neg. / neg.**	**-1.593**	**-1.049**	253 (121)
**Canine size dimorphism**		**neg. / neg.**	**-2.278**	**-1.485**	163 (66)
**Adult sex ratio (M/(F+M))**		**pos. / pos.**	**0.844**	**1.400**	235 (112)
**Male reproductive skew**		**neg. / neg.**	**-0.773**	**-0.062**	71 (19)
Relative testis mass		pos. / none	-0.579	0.150	141 (52)
Receptive synchrony		pos. / neg.	**-0.973**	**-0.127**	104 (38)
(2) Quantitative response variable (% contests won by females), full sample					
**Mating system**	**Monogamy vs. polygyny**	**pos. / pos.**	**0.496**	**0.819**	144 (79)
	**Monogamy vs. polygynandry**	**pos. / pos.**	**0.685**	**0.854**	
	Monogamy vs. polyandry	none / pos.	0.171	0.568	
	Polygynandry vs polygyny	pos. / none	-0.272	0.037	
	**Polyandry vs polygyny**	**pos. / pos.**	**0.089**	**0.523**	
	**Polyandry vs polygynandry**	**pos. / pos.**	**0.239**	**0.594**	
**Substrate use**	**Arboreal vs. terrestrial**	**pos. / pos.**	**0.811**	**0.947**	150 (83)
	**Arboreal vs. scansorial**	**pos. / pos.**	**0.536**	**0.807**	
	**Terrestrial vs. scansorial**	**neg. / neg.**	**-0.373**	**-0.063**	
**Length of sexual receptivity**		**neg. / neg.**	**-2.386**	**-1.591**	64 (29)
**Sexual size dimorphism**		**neg. / neg.**	**-2.896**	**-2.209**	151 (84)
**Canine size dimorphism**		**neg. / neg.**	**-2.359**	**-1.581**	97 (50)
**Adult sex ratio (M/(F+M))**		**pos. / pos.**	**0.612**	**0.854**	145 (81)
Male reproductive skew		neg. / pos.	0.213	0.960	46 (18)
**Relative testis mass**		**pos. / pos.**	**1.149**	**1.948**	126 (51)
Receptive synchrony		pos. / neg.	-2.140	-1.026	60 (32)

Supplementary Table S2: Summary of the phylogenetically-controlled tests of the 'reproductive control' hypothesis, excluding lemurs from the sample. These extra models were run to test if significant results do not simply capture general contrasts between lemurs and non-lemurs. The direction of expected (Exp.) and observed (Obs.) associations is compared to assess support for each prediction; results in support of the predictions are highlighted in bold. The confidence interval of significant associations does not include 0.

Explanatory variables		Exp. / Obs.	CI Estimate		N. obs. (N. sp.)
			Lower	Upper	
(1) Categorical response variable (strict male dominance, no strict bias in dominance, strict female dominance), lemurs excluded					
**Mating system**	**Monogamy vs. polygyny**	**pos. / pos.**	**0.150**	**0.502**	185 (84)
	Monogamy vs. polygynandry	pos. / none	-0.010	0.356	
	**Monogamy vs. polyandry**	**none / none**	-0.160	0.266	
	**Polygynandry vs polygyny**	**pos. / pos.**	**0.029**	**0.290**	
	**Polyandry vs polygyny**	**pos. / pos.**	**0.090**	**0.454**	
	**Polyandry vs polygynandry**	pos. / none	-0.05	0.293	
**Substrate use**	**Arboreal vs. Terrestrial**	**pos. / pos.**	**0.050**	**0.361**	185 (82)
	**Arboreal vs. Scansorial**	**pos. / pos.**	**0.000**	**0.256**	
	Terrestrial vs. Scansorial	neg. / none	-0.230	0.087	
Length of sexual receptivity		neg. / none	-0.300	0.500	86 (29)
**Sexual size dimorphism**		**neg. / neg.**	**-0.895**	**-0.340**	189 (85)
**Canine size dimorphism**		**neg. / neg.**	**-1.606**	**-0.740**	130 (51)
**Adult sex ratio (M/(F+M))**		**pos. / pos.**	**0.359**	**0.880**	172 (77)
Male reproductive skew		neg. / none	-0.873	0.040	57 (17)
Relative testis mass		pos. / none	-0.526	0.130	116 (42)
Receptive synchrony		pos. / none	-0.579	0.240	91 (31)
(2) Quantitative response variable (% contests won by females), lemurs excluded					
**Mating system**	**Monogamy vs. polygyny**	**pos. / pos.**	**0.000**	**0.637**	95 (51)
	Monogamy vs. polygynandry	pos. / none	-0.210	0.467	
	**Monogamy vs. polyandry**	**none /none**	**-0.420**	**0.395**	
	**Polygynandry vs polygyny**	**pos. / pos.**	**0.034**	**0.360**	
	**Polyandry vs polygyny**	**pos. / pos.**	**0.060**	**0.606**	
	Polyandry vs polygynandry	pos. / none	-0.110	0.395	
**Substrate use**	**Arboreal (vs. terrestrial)**	**pos. / pos.**	**0.280**	**0.663**	94 (50)
	**Arboreal (vs. scansorial)**	**pos. / pos.**	**0.230**	**0.605**	
	Terrestrial (vs. scansorial)	neg. / none	-0.180	0.070	
Length of sexual receptivity		neg. / none	-0.022	0.920	49 (25)
**Sexual size dimorphism**		**neg. / neg.**	**-1.067**	**-0.350**	95 (51)
**Canine size dimorphism**		**neg. / neg.**	**-0.995**	**-0.050**	70 (37)
**Adult sex ratio (M/(F+M))**		**pos. / pos.**	**0.404**	**0.640**	90 (49)
**Male reproductive skew**		**neg. / neg.**	**-1.488**	**-0.880**	36 (16)
Relative testis mass		pos. / neg.	-0.990	-0.010	58 (29)
Receptive synchrony		pos. / neg.	-1.435	-0.710	49 (25)

Supplementary Table S3: Summary of the phylogenetically controlled tests of the female competition hypothesis in the full sample. The direction of expected (Exp.) and observed (Obs.) associations is compared to assess support for each prediction; results in support of the predictions are highlighted in bold. The confidence interval of significant associations does not include 0.

	Explanatory variable	Exp. / Obs.	CI Estimate		N. obs. (N. sp.)
			Lower	Upper	
(1) Categorical response variable (strict male dominance, no strict bias in dominance, strict female dominance), full sample					
Social organization	**Solitary vs. group-living**	**pos. / pos.**	**0.166**	**0.485**	226 (117)
	**Pair-living vs. group-living**	**pos. / pos.**	**0.236**	**0.530**	
	**Pair-living vs. solitary**	**none / none**	**-0.134**	**0.254**	
Social competition	**Fission-fusion system (vs stable group)**	**neg. / neg.**	**-0.471**	**-0.206**	253 (121)
	**Home range overlap**	**neg. / neg.**	**-0.898**	**-0.317**	150 (63)
	**Number of females in group**	**neg. / neg.**	**-1.119**	**-0.578**	236 (113)
	**Female evictions (vs. no evictions)**	**pos. / pos.**	**0.245**	**0.519**	101 (33)
	Female infanticide (vs. no infanticide)	pos. / none	-0.115	0.191	153 (63)
	Female relative canine size	pos. / neg.	-0.919	-0.208	153 (59)
Resource competition	Natural population (vs. captive)	pos. / none	-0.166	0.048	253 (121)
	Environmental harshness	pos. / neg.	-0.943	-0.246	135 (68)
	**Rainfall seasonality**	**pos. / pos.**	**0.006**	**0.665**	134 (69)
	Rainfall unpredictability	pos. / neg.	-1.008	-0.342	135 (68)
(2) Quantitative response variable (% contests won by females), full sample					
Social organization	**Solitary vs. Group-living**	**pos. / pos.**	**0.518**	**0.808**	151 (84)
	**Pair-living vs. Group-living**	**pos. / pos.**	**0.699**	**0.864**	
	**Pair-living vs. solitary**	**none / none**	**-0.028**	**0.290**	
Social competition	**Fission-fusion system (vs stable group)**	**neg. / neg.**	**-0.753**	**-0.505**	151 (84)
	Home range overlap	neg. / none	-0.413	0.151	99 (48)
	**Number of females in group**	**neg. / neg.**	**-0.784**	**-0.482**	146 (82)
	**Female evictions (vs. no evictions)**	**pos. / pos.**	**0.574**	**0.771**	58 (25)
	Female infanticide (vs. no infanticide)	pos. / none	-0.071	0.192	93 (45)
	Female relative canine size	pos. / neg.	-0.713	-0.099	89 (43)
Resource competition	**Natural population (vs. captive)**	**pos. / pos.**	**0.190**	**0.343**	151 (84)
	Environmental harshness	pos. / none	-0.537	0.254	86 (48)
	Rainfall seasonality	pos. / none	-0.471	0.562	85 (48)
	**Rainfall unpredictability**	**pos. / pos.**	**0.054**	**0.907**	**86 (48)**

Supplementary Table S4: Summary of the phylogenetically-controlled tests of the 'female competition' hypothesis, excluding lemurs from the sample. These extra models were run to test if significant results do not simply capture general contrasts between lemurs and non-lemurs. The direction of expected (Exp.) and observed (Obs.) associations is compared to assess support for each prediction; results in support of the predictions are highlighted in bold. The confidence interval of significant associations does not include 0.

	Explanatory variable	Exp. / Obs.	CI Estimate		N. obs. (N. sp.)
			Lower	Upper	
(1) Categorical response variable (strict male dominance, no strict bias in dominance, strict female dominance), lemurs excluded					
Social organization	Solitary vs. group-living	No variation in sample subset			
	**Pair-living vs. group-living**	**pos. / pos.**	**0.057**	**0.450**	172 (83)
	Pair-living vs. solitary	No variation in sample subset			
Social competition	**Fission-fusion system (vs stable group)**	**neg. / neg.**	**-0.280**	**-0.001**	189 (85)
	Home range overlap	neg. / none	-0.352	0.250	118 (51)
	**Number of females in group**	**neg. / neg.**	**-0.631**	**-0.090**	172 (77)
	Female evictions (vs. no evictions)	pos. / none	-0.220	0.190	84 (28)
	Female infanticide (vs. no infanticide)	pos. / none	-0.145	0.150	125 (52)
	Female relative canine size	pos. / neg.	-0.785	-0.010	124 (48)
Resource competition	Natural population (vs. captive)	pos. / none	-0.163	0.060	189 (85)
	Environmental harshness	pos. / none	-0.705	0.020	107 (51)
	Rainfall seasonality	pos. / none	-0.322	0.520	105 (51)
	Rainfall unpredictability	pos. / none	-0.395	0.360	107 (15)
(2) Quantitative response variable (% contests won by females), lemurs excluded					
Social organization	Solitary vs. group-living	No variation in sample subset			
	Pair-living vs. group-living	pos. / none	-0.103	0.570	95 (51)
	Pair-living vs. solitary	No variation in sample subset			
Social competition	**Fission-fusion system (vs stable group)**	**neg. / neg.**	**-0.592**	**-0.170**	95 (51)
	**Home range overlap**	**neg. / neg.**	**-0.741**	**-0.110**	73 (38)
	**Number of females in group**	**neg. / neg.**	**-0.689**	**-0.360**	90 (49)
	**Female evictions (vs. no evictions)**	**pos. / pos.**	**0.268**	**0.590**	47 (22)
	**Female infanticide (vs. no infanticide)**	**pos. / pos.**	**0.092**	**0.440**	71 (36)
	Female relative canine size	pos. / none	-0.753	0.320	66 (34)
Resource competition	**Natural population (vs. captive)**	**pos. / pos.**	**0.071**	**0.230**	95 (51)
	**Environmental harshness**	**pos. / pos.**	**0.230**	**1.020**	61 (32)
	Rainfall seasonality	pos. / none	-0.404	0.170	59 (31)
	**Rainfall unpredictability**	**pos. / pos.**	**0.688**	**1.400**	61 (32)

Supplementary Table S5: Summary of the phylogenetically-controlled tests of predictions for the offspring safety hypothesis. In addition of the main models (1) and (2) below, two extra models (3) and (4) were run in a sample subset excluding lemurs to test if significant results do not simply capture general contrasts between lemurs and non-lemurs. The direction of expected (Exp.) and observed (Obs.) associations is compared to assess support for each prediction; results in support of the predictions are highlighted in bold. The confidence interval of significant associations does not include 0.

Explanatory variable	Exp / Obs	CI Estimate		N. obs.
		Lower	Upper	(N. sp.)
(1) Categorical response variable (strict male dominance, no strict sex bias, strict female dominance), full sample				
**Infant parking (vs. carrying)**	**pos. / pos.**	**0.269**	**0.545**	253 (121)
**Relative lactation duration**	**neg. / neg.**	**-1.336**	**-0.687**	154 (63)
**Infanticide by males (yes / no)**	**neg. / neg.**	**-0.277**	**-0.004**	146 (57)
Allomaternal care (yes / no)	pos. / none	-0.026	0.273	206 (97)
(2) Quantitative response variable (% contests won by females), full sample				
**Infant parking (vs. carrying)**	**pos. / pos.**	**0.316**	**0.660**	151 (84)
**Relative lactation duration**	**neg. / neg.**	**-1.178**	**-0.612**	97 (49)
Infanticide by males (yes / no)	neg. / none	-0.007	0.304	83 (41)
Allomaternal care (yes / no)	pos. / none	-0.306	0.082	125 (68)
(3) Categorical response variable (strict male dominance, no strict sex bias, strict female dominance), lemurs excluded				
Infant parking (vs. carrying)		No variation in sample subset		
Relative lactation duration	neg. / none	-0.631	0.021	124 (52)
**Infanticide by males (yes / no)**	**neg. / neg.**	**-0.469**	**-0.233**	123 (49)
**Allomaternal care (yes / no)**	**pos. / pos.**	**0.128**	**0.430**	160 (73)
(4) Quantitative response variable (% contests won by females), lemurs excluded				
Infant parking (vs. carrying)		No variation in sample subset		
Relative lactation duration	neg. / none	-0.400	0.288	73 (40)
Infanticide by males (yes / no)	neg. / none	-0.269	0.024	66 (35)
**Allomaternal care (yes / no)**	**pos. / pos.**	**0.348**	**0.657**	85 (46)

Supplementary Table S6: Summary of phylogenetically-controlled tests investigating predictions for the social bonds hypothesis. In addition of the main models (1) and (2) below, two extra models (3) and (4) were run in a subset of species with moderate sexual size dimorphism (i.e., where male body mass is lower than female body mass*1.10) to test if the social bonds hypothesis may only be detectable when its effects are not overridden by a large sexual size dimorphism. The direction of expected (Exp.) and observed (Obs.) associations is compared to assess support for each prediction; results in support of the predictions are highlighted in bold. The confidence interval of significant associations does not include 0.

Explanatory variable		Exp / Obs	CI Estimate		N. obs. (N. sp.)
			Lower	Upper	
(1) Categorical response variable (strict male dominance, no strict sex bias, strict female dominance), full sample					
	**Female (vs male)**	**pos. / pos.**	**0.074**	**0.384**	
**Philopatric sex**	Female (vs both sexes disperse)	pos. / none	-0.091	0.219	153 (66)
	Male (vs both sexes disperse)	neg. / none	-0.334	0.023	
Female average relatedness in groups		pos. / none	-0.283	0.618	51 (16)
Female-female coalitions (vs. no coalitions)		pos. / neg.	-0.283	-0.017	210 (99)
(2) Quantitative response variable (% contests won by females), full sample					
	**Female (vs male)**	**pos. / pos.**	**0.000**	**0.343**	
**Philopatric sex**	Female (vs both sexes disperse)	pos. / none	-0.207	0.047	94 (47)
	**Male (vs both sexes disperse)**	**neg. / neg.**	**-0.416**	**-0.078**	
Female average relatedness in groups		pos. / neg.	-1.665	-0.304	39 (14)
Female-female coalitions (vs. no coalitions)		pos. / neg.	-0.551	-0.224	127 (69)
(3) Categorical response variable (strict male dominance, no strict sex bias, strict female dominance), subset of species with low sexual size dimorphism					
	**Female (vs male)**	**pos. / pos.**	**0.176**	**0.563**	
**Philopatric sex**	**Female (vs both sexes disperse)**	**pos. / pos.**	**0.149**	**0.479**	50 (19)
	Male (vs both sexes disperse)	neg. / none	-0.253	0.141	
Female average relatedness in groups		pos. / none	-1.290	0.126	24 (7)
Female-female coalitions (vs. no coalitions)		pos. / none	-0.112	0.235	72 (37)
(4) Quantitative response variable (% contests won by females), subset of species with low sexual size dimorphism					
	**Female (vs male)**	**pos. / pos.**	**0.224**	**0.798**	
**Philopatric sex**	**Female (vs both sexes disperse)**	**pos. / pos.**	**0.171**	**0.594**	36 (14)
	Male (vs both sexes disperse)	neg. / none	-0.470	0.174	
Female average relatedness in groups		pos. / none	-0.621	0.307	20 (6)
Female-female coalitions (vs. no coalitions)		pos. / neg.	-0.826	-0.609	55 (29)

Supplementary Table S7: Summary of the phylogenetically-controlled tests of predictions for the self-organization hypothesis. In addition of the main models (1) and (2) below, two extra models (3) and (4) were run in a subset of species with moderate sexual size dimorphism (i.e., where male body mass is lower than female body mass*1.10) to test if the self-organization hypothesis may only be detectable when its effects are not overridden by a large sexual size dimorphism. The direction of expected (Exp.) and observed (Obs.) associations is compared to assess support for each prediction; results in support of the predictions are highlighted in bold. The confidence interval of significant associations does not include 0.

Explanatory variable	Exp / Obs	CI Estimate		N. obs. (N. sp.)
		Lower	Upper	
**(1) Categorical response variable (strict male dominance, no strict sex bias, strict female dominance), full sample**				
**Adult sex ratio (M/(F+M))**	**pos. / pos.**	**0.844**	**1.400**	235 (112)
**Percentage of male-male contests**	**pos. / pos.**	**0.398**	**1.108**	91 (51)
Number of males in group	pos. / none	-0.266	0.216	236 (113)
**(2) Quantitative response variable (% contests won by females), full sample**				
**Adult sex ratio (M/(F+M))**	**pos. / pos.**	**0.612**	**0.854**	145 (81)
Percentage of male-male contests	pos. / neg.	-0.347	-0.111	81 (45)
Number of males in group	pos. / none	-0.004	0.217	146 (82)
**(3) Categorical response variable (strict male dominance, moderate sex bias, strict female dominance), subset of species with low sexual size dimorphism**				
**Adult sex ratio (M/(F+M))**	**pos. / pos.**	**0.408**	**1.234**	89 (49)
**Percentage of male-male contests**	**pos. / pos.**	**0.309**	**1.484**	34 (17)
Number of males in group	pos. / none	-0.125	0.708	90 (50)
**(4) Quantitative response variable (% contests won by females), subset of species with low sexual size dimorphism**				
**Adult sex ratio (M/(F+M))**	**pos. / pos.**	**0.680**	**1.011**	70 (40)
Percentage of male-male contests	pos. / none	-0.261	0.081	30 (16)
Number of males in group	pos. / none	-0.264	0.046	72 (41)

Supplementary Table S8: Original data with references.

Species	Classification of intersexual dominance	Percent fights won by females	Percent of all aggression that is F-F	F-M	M-M	Reference
Alouatta caraya	strict male dominance	5	51	49	NA	Jones, C. B. (1983). Social organization of captive black howler monkeys (Alouatta caraya):“social competition” and the use of non-damaging behavior. Primates, 24(1), 25-39.
Alouatta palliata	strict male dominance	0	41	52	7	Jones, C. B. (1980). The functions of status in the mantled howler monkey, Alouatta palliata Gray: intraspecific competition for group membership in a folivorous neotropical primate. Primates, 21(3), 389-405.
Alouatta palliata	strict male dominance	0	33	63	4	Jones, C. B. (1980). The functions of status in the mantled howler monkey, Alouatta palliata Gray: intraspecific competition for group membership in a folivorous neotropical primate. Primates, 21(3), 389-405.
Alouatta palliata	strict male dominance	0	13	87	0	Larose F. 1996. Foraging strategies, group size, and foodcompetition in the mantled howler monkey,Alouatapalliata. Ph.D. thesis. University of Alberta, Edmonton,Canada.
Alouatta palliata	strict male dominance	0	NA	NA	NA	Wang, E., & Milton, K. (2003). Intragroup social relationships of male Alouatta palliata on Barro Colorado Island, Republic of Panama. International Journal of Primatology, 24(6), 1227-1243.

Alouatta pigra	strict male dominance	NA	54	44	2	Van Belle, S., Estrada, A., & Di Fiore, A. (2014). Kin-biased spatial associations and social interactions in male and female black howler monkeys (Alouatta pigra). Behaviour, 151(14), 2029-2057.
Alouatta seniculus	no strict sex bias in dominance	NA	42	33	25	Rimbach, R (2013) Effects of forest fragmentation on brown spider monkeys (Ateles hybridus) and red howler monkeys (Alouatta seniculus) PhD thesis Göttingen Germany
Aotus azarae	no strict sex bias in dominance	37	NA	NA	NA	Corley, M. K., Xia, S., & Fernandez_Duque, E. (2017). The role of intragroup agonism in parent_offspring relationships and natal dispersal in monogamous owl monkeys (Aotus azarae) of Argentina. American journal of primatology, 79(11), e22712.
Ateles belzebuth	strict male dominance	6	84	10	6	Gibson, K. N. (2008). Mating tactics and socioecology of male white-bellied spider monkeys (Ateles belzebuth chamek). Yale University.
Ateles belzebuth	no strict sex bias in dominance	42	60	37	3	van de Wassenberg, B. Social behaviour of Peruvian spider monkeys (Ateles chamek) during the pre-release stage.
Ateles geoffroyi	strict male dominance	0	87	13	0	Asensio, N., Aureli, F., Schaffner, C., & Korstjens, A. (2008). Intragroup aggression, fission–fusion dynamics and feeding competition in spider monkeys. Behaviour, 145(7), 983-1001.
Ateles geoffroyi	strict male dominance	0	NA	NA	NA	Fedigan, L. M., & Baxter, M. J. (1984). Sex differences and social organization in free-ranging spider monkeys (Ateles geoffroyi). Primates, 25(3), 279-294.

Ateles geoffroyi	strict male dominance	1	92	3	5	Slater, K. Y., Schaffner, C. M., & Aureli, F. (2009). Sex differences in the social behavior of wild spider monkeys (Ateles geoffroyi yucatanensis). American Journal of Primatology: Official Journal of the American Society of Primatologists, 71(1), 21-29.
Ateles hybridus	strict male dominance	0	50	39	11	Rimbach, R., Link, A., Montes_Rojas, A., Di Fiore, A., Heistermann, M., & Heymann, E. W. (2014). Behavioral and physiological responses to fruit availability of spider monkeys ranging in a small forest fragment. American journal of primatology, 76(11), 1049-1061.
Avahi occidentalis	strict female dominance	100	NA	NA	NA	Ramanankirahina, R., Joly, M., & Zimmermann, E. (2011). Peaceful primates: affiliation, aggression, and the question of female dominance in a nocturnal pair_living lemur (Avahi occidentalis). American Journal of Primatology, 73(12), 1261-1268.
Brachyteles arachnoides	no strict sex bias in dominance	60	25	35	40	Strier, K. B. (1992). Causes and consequences of nonaggression in the woolly spider monkey, or muriqui (Brachyteles arachnoides). Aggression and peacefulness in humans and other primates, 100-116.
Cacajao calvus	no strict sex bias in dominance	NA	0	0	100	Bowler, M., & Bodmer, R. (2009). Social behavior in fission–fusion groups of red uakari monkeys (Cacajao calvus ucayalii). American Journal of Primatology, 71(12), 976-987.
Cacajao melanocephalus	no strict sex bias in dominance	NA	NA	NA	NA	Barnett, A. A., Bowler, M., Bezerra, B. M., & Defler, T. R. (2013). Ecology and behavior of uacaris (genus Cacajao). Evolutionary biology and conservation of titis, sakis and uacaris, 151-172.
Cacajao ouakary	no strict sex bias in dominance	NA	0	100	0	Barnett, A. A. (2010). Diet, habitat, use and conservation ecology of the golden-backed uacari, Cacajao melanocephalus ouakary, in Jau_ National Park, Amazonian Brazil (Doctoral dissertation, University of Roehampton).

Cacajao ouakary	no strict sex bias in dominance	NA	NA	NA	NA	Barnett, A. A., Bowler, M., Bezerra, B. M., & Defler, T. R. (2013). Ecology and behavior of uacaris (genus Cacajao). Evolutionary biology and conservation of titis, sakis and uacaris, 151-172.
Callicebus coimbrai	no strict sex bias in dominance	NA	NA	NA	NA	Souza-Alves, J. P., Chagas, R. R., & Bezerra, B. M. (2019). Food-sharing behaviour within a group of free-living Endangered Coimbra-Filho's titi monkeys. Journal of Ethology, 37(1), 41-46.
Callicebus cupreus	no strict sex bias in dominance	NA	NA	NA	NA	Dolotovskaya, S., Walker, S., & Heymann, E. W. (2020). What makes a pair bond in a Neotropical primate: female and male contributions. Royal Society Open Science, 7(1), 191489.
Callicebus moloch	no strict sex bias in dominance	NA	NA	NA	NA	Anzenberger, G. (1988). The pairbond in the titi monkey (Callicebus moloch): intrinsic versus extrinsic contributions of the pairmates. Folia Primatologica, 50(3-4), 188-203.
Callithrix flaviceps	no strict sex bias in dominance	NA	91	9	0	Ferrari SF. 2009. Social Hierarchy and Dispersal in Free-Ranging Buffy-Headed Marmosets (Callithrix flaviceps). In: Ford SM, Porter LM, Davis LC, editors. The Smallest Anthropoids: The Marmoset/Callimico Radiation. Boston, MA: Springer US. p 155-165.
Callithrix jacchus	no strict sex bias in dominance	84	62	24	14	De la Fuente MF, Schiel N, Bicca-Marques JC, Caselli CB, Souto A, Garber PA (2019) Balancing contest competition, scramble competition, and social tolerance at feeding sites in wild common marmosets (Callithrix jacchus). American Journal of Primatology 81:e22964
Callithrix jacchus	no strict sex bias in dominance	47	73	25	2	Digby L (1995) Social organization in a wild population of Callithrix jacchus. II. Intragroup social behavior. Primates 36:361-375

Callithrix penicillata	no strict sex bias in dominance	38	78	15	7	Decanini DP, Macedo RH. 2008. Sociality in Callithrix penicillata: I. Intragroup Male Profile. International Journal of Primatology 29:433-447.
Cebuella pygmaea	strict female dominance	NA	NA	NA	NA	Soini, P. (1987). Sociosexual behavior of a free_ranging Cebuella pygmaea (Callitrichidae, platyrrhini) troop during postpartum estrus of its reproductive female. American Journal of Primatology, 13(3), 223-230.
Cebus albifrons	no strict sex bias in dominance	44	45	23	32	Negrette Bohórquez, K. J. (2015). The social behavior of white-fronted capuchin monkey (Cebus albifrons versicolor) and the case of an alpha male disappearance at San Juan de Carare, Colombia (Bachelor's thesis, Bogotá-Uniandes).
Cebus capucinus	no strict sex bias in dominance	31	53	16	31	Fedigan, L. (1993). Sex differences and intersexual relations in adult white-faced capuchins (Cebus capucinus). International Journal of Primatology, 14(6), 853-877.
Cebus capucinus	no strict sex bias in dominance	27	55	20	25	Fedigan, L. (1993). Sex differences and intersexual relations in adult white-faced capuchins (Cebus capucinus). International Journal of Primatology, 14(6), 853-877.
Cebus capucinus	no strict sex bias in dominance	NA	NA	NA	NA	Leca, J. B., Fornasieri, I., & Petit, O. (2002). Aggression and reconciliation in Cebus capucinus. International Journal of Primatology, 23(5), 979-998.
Cebus capucinus	strict male dominance	3	NA	NA	NA	Perry, S. (1997). Male-female social relationships in wild white-faced capuchins (Cebus capucinus). Behaviour, 134(7), 477-510.

Cebus olivaceus	no strict sex bias in dominance	57	NA	NA	NA	O'Brien, T. G. (1991). Female-male social interactions in wedge-capped capuchin monkeys: benefits and costs of group living. Animal Behaviour, 41(4), 555-567.
Cercocebus atys	strict male dominance	NA	NA	NA	NA	Bernstein (1976) Activity Patterns in a Sooty Mangabey Group
Cercocebus atys	strict male dominance	NA	NA	NA	NA	Stahl et al (2003) Food competition in captive female sooty mangabeys(Cercocebus torquatus atys)
Cercocebus atys	strict male dominance	NA	NA	NA	NA	Stahl et al (2003) Food competition in captive female sooty mangabeys(Cercocebus torquatus atys)
Cercocebus atys	strict male dominance	NA	NA	NA	NA	Dolado et al (2013) Agonistic Strategies and Spatial Distribution in Captive Sooty Mangabeys (Cercocebus Atys)
Cercocebus torquatus	strict male dominance	NA	NA	NA	NA	Dolado et al (2011) Dominance hierarchy and spatial distribution in captive red-capped mangabeys (Cercocebus torquatus torquatus)
Cercopithecus campbelli	strict male dominance	NA	NA	NA	NA	Lemasson et (2006) Female Social Relationships in a Captive Group of Campbell’s Monkeys (Cercopithecus campbelli campbelli)

Cercopithecus diana	no strict sex bias in dominance	24	NA	NA	NA	Byrne et al (1983) Social Relationships in a Captive Group of Diana Monkeys (Cercopithecus diana)
Cercopithecus petaurista	strict male dominance	2	NA	NA	NA	Reinhart (2019)The Effect of Social Hierarchy on Behavior inCercopithecus petaurista
Cercopithecus solatus	no strict sex bias in dominance	NA	NA	NA	NA	Peignot et al (2002) A Preliminary Study on the Social Relationships in a Semi-free Ranging Colony of Sun-tailed Monkeys (Cercopithecus solatus), a Species Recently Discovered in Gabon
Chiropotes sagulatus	no strict sex bias in dominance	NA	NA	NA	NA	Gregory, L. T. (2011). Socioecology of the Guianan bearded saki, Chiropotes sagulatus (Doctoral dissertation, Kent State University).
Chiropotes sagulatus	no strict sex bias in dominance	NA	NA	NA	NA	Gregory, L. T. (2011). Socioecology of the Guianan bearded saki, Chiropotes sagulatus (Doctoral dissertation, Kent State University).
Chlorocebus pygerythrus	no strict sex bias in dominance	NA	NA	NA	NA	Hemelrijk et al (2020) Dynamics of Intersexual Dominance and Adult Sex- Ratio in Wild Vervet Monkeys
Chlorocebus pygerythrus	no strict sex bias in dominance	NA	NA	NA	NA	Hemelrijk et al (2020) Dynamics of Intersexual Dominance and Adult Sex- Ratio in Wild Vervet Monkeys

Chlorocebus pygerythrus	no strict sex bias in dominance	NA	NA	NA	NA	Hemelrijk et al (2020) Dynamics of Intersexual Dominance and Adult Sex- Ratio in Wild Vervet Monkeys
Chlorocebus pygerythrus	no strict sex bias in dominance	NA	NA	NA	NA	Hemelrijk et al (2020) Dynamics of Intersexual Dominance and Adult Sex- Ratio in Wild Vervet Monkeys
Chlorocebus pygerythrus	no strict sex bias in dominance	NA	NA	NA	NA	Hemelrijk et al (2020) Dynamics of Intersexual Dominance and Adult Sex- Ratio in Wild Vervet Monkeys
Chlorocebus pygerythrus	no strict sex bias in dominance	NA	NA	NA	NA	Hemelrijk et al (2020) Dynamics of Intersexual Dominance and Adult Sex- Ratio in Wild Vervet Monkeys
Chlorocebus pygerythrus	strict male dominance	NA	NA	NA	NA	Hemelrijk et al (2020) Dynamics of Intersexual Dominance and Adult Sex- Ratio in Wild Vervet Monkeys
Chlorocebus pygerythrus	no strict sex bias in dominance	NA	NA	NA	NA	Hemelrijk et al (2020) Dynamics of Intersexual Dominance and Adult Sex- Ratio in Wild Vervet Monkeys
Chlorocebus pygerythrus	no strict sex bias in dominance	NA	NA	NA	NA	Hemelrijk et al (2020) Dynamics of Intersexual Dominance and Adult Sex- Ratio in Wild Vervet Monkeys

Chlorocebus pygerythrus	no strict sex bias in dominance	20	33	39	28	Kappeler P.M., Huchard E., Baniel A., Canteloup C., Charpentier M.J.E., Cheng L., Davidian E., Duboscq J., Fichtel C., Hemelrijk C.K., Hoener O.P., Koren L., Micheletta J., Prox L., Saccà T., Seex L., Smit N., Surbeck M., van de Waal E., Girard-Buttoz C. (2022) Sex and dominance: How to assess and interpret intersexual dominance relationships in mammalian societies, Frontiers in Ecology and Evolution 10, 10.3389/fevo.2022.918773. (Table S4, Index 5)
Chlorocebus pygerythrus	no strict sex bias in dominance	30	37	17	46	Kappeler P.M., Huchard E., Baniel A., Canteloup C., Charpentier M.J.E., Cheng L., Davidian E., Duboscq J., Fichtel C., Hemelrijk C.K., Hoener O.P., Koren L., Micheletta J., Prox L., Saccà T., Seex L., Smit N., Surbeck M., van de Waal E., Girard-Buttoz C. (2022) Sex and dominance: How to assess and interpret intersexual dominance relationships in mammalian societies, Frontiers in Ecology and Evolution 10, 10.3389/fevo.2022.918773. (Table S4, Index 5)
Chlorocebus pygerythrus	no strict sex bias in dominance	40	13	55	32	Kappeler P.M., Huchard E., Baniel A., Canteloup C., Charpentier M.J.E., Cheng L., Davidian E., Duboscq J., Fichtel C., Hemelrijk C.K., Hoener O.P., Koren L., Micheletta J., Prox L., Saccà T., Seex L., Smit N., Surbeck M., van de Waal E., Girard-Buttoz C. (2022) Sex and dominance: How to assess and interpret intersexual dominance relationships in mammalian societies, Frontiers in Ecology and Evolution 10, 10.3389/fevo.2022.918773. (Table S4, Index 5)
Chlorocebus pygerythrus	no strict sex bias in dominance	19	25	69	6	Kappeler P.M., Huchard E., Baniel A., Canteloup C., Charpentier M.J.E., Cheng L., Davidian E., Duboscq J., Fichtel C., Hemelrijk C.K., Hoener O.P., Koren L., Micheletta J., Prox L., Saccà T., Seex L., Smit N., Surbeck M., van de Waal E., Girard-Buttoz C. (2022) Sex and dominance: How to assess and interpret intersexual dominance relationships in mammalian societies, Frontiers in Ecology and Evolution 10, 10.3389/fevo.2022.918773. (Table S4, Index 5)
Daubentonia madagascariensis	strict female dominance	68	NA	NA	NA	Rendall, D. (1993). Does female social precedence characterize captive aye-ayes (Daubentonia madagascariensis)?. International journal of primatology, 14, 125-130.

Erythrocebus patas	no strict sex bias in dominance	20	NA	NA	NA	Hall, K. R. L., & Mayer, B. (1967). SOCIAL INTERACTIONS IN A GROUP OF CAPTIVE PATAS MONKEYS (ERYTHROCEBUS PATAS). Folia Primatologica, 5(3), 213–236.doi:10.1159/000161948
Erythrocebus patas	strict male dominance	0	2	98	0	Kaplan, J. R., & Zucker, E. (1980). Social Organization in a Group of Free-Ranging Patas Monkeys. Folia Primatologica, 34(3-4), 196–213. doi:10.1159/000155955
Erythrocebus patas	no strict sex bias in dominance	61	NA	NA	NA	Hall, K. R. L. (2009). Behaviour and ecology of the wild Patas monkey, Erythrocebus patas, in Uganda. Journal of Zoology, 148(1), 15–87.doi:10.1111/j.1469-7998.1966.tb02942.x
Erythrocebus patas	no strict sex bias in dominance	3	NA	NA	NA	Goldman, E. N., & Loy, J. (1997). Longitudinal study of dominance relations among captive patas monkeys. American Journal of Primatology, 42(1), 41–51.doi:10.1002/(sici)1098-2345(1997)42:13.0.co;2-z
Eulemur coronatus	no strict sex bias in dominance	88	NA	NA	NA	Marolf et al. 2007
Eulemur flavifrons	strict female dominance	99	91	8	1	Digby&Kahlenberg 2002; Digby & Stevens 2007
Eulemur fulvus	no strict sex bias in dominance	NA	NA	NA	NA	Roeder et al. 2002

Eulemur macaco	no strict sex bias in dominance	NA	NA	NA	NA	Roeder et al. 2002
Eulemur rubriventer	no strict sex bias in dominance	47	NA	NA	NA	Marolf et al. 2007
Eulemur rufifrons	no strict sex bias in dominance	NA	68	19	13	Pereira & Kappeler 1996
Eulemur rufifrons	no strict sex bias in dominance	21	55	6	39	Kappeler P.M., Huchard E., Baniel A., Canteloup C., Charpentier M.J.E., Cheng L., Davidian E., Duboscq J., Fichtel C., Hemelrijk C.K., Hoener O.P., Koren L., Micheletta J., Prox L., Saccà T., Seex L., Smit N., Surbeck M., van de Waal E., Girard-Buttoz C. (2022) Sex and dominance: How to assess and interpret intersexual dominance relationships in mammalian societies, Frontiers in Ecology and Evolution 10, 10.3389/fevo.2022.918773. (Table S4, Index 5)
Eulemur rufifrons	no strict sex bias in dominance	14	35	2	63	Kappeler P.M., Huchard E., Baniel A., Canteloup C., Charpentier M.J.E., Cheng L., Davidian E., Duboscq J., Fichtel C., Hemelrijk C.K., Hoener O.P., Koren L., Micheletta J., Prox L., Saccà T., Seex L., Smit N., Surbeck M., van de Waal E., Girard-Buttoz C. (2022) Sex and dominance: How to assess and interpret intersexual dominance relationships in mammalian societies, Frontiers in Ecology and Evolution 10, 10.3389/fevo.2022.918773. (Table S4, Index 5)
Eulemur rufifrons	no strict sex bias in dominance	40	73	9	18	Kappeler P.M., Huchard E., Baniel A., Canteloup C., Charpentier M.J.E., Cheng L., Davidian E., Duboscq J., Fichtel C., Hemelrijk C.K., Hoener O.P., Koren L., Micheletta J., Prox L., Saccà T., Seex L., Smit N., Surbeck M., van de Waal E., Girard-Buttoz C. (2022) Sex and dominance: How to assess and interpret intersexual dominance relationships in mammalian societies, Frontiers in Ecology and Evolution 10, 10.3389/fevo.2022.918773. (Table S4, Index 5)

Gorilla beringei	strict male dominance	0	80	18	2	Robbins, M. M. (2008). Feeding competition and agonistic relationships among Bwindi Gorilla beringei. International Journal of Primatology, 29(4), 999.
Gorilla beringei	strict male dominance	1	59	32	9	Watts, D. P. (1992). Social relationships of immigrant and resident female mountain gorillas. I. Male_female relationships. American journal of primatology, 28(3), 159-181.
Gorilla beringei	strict male dominance	1	NA	NA	NA	Watts, D. P. (1992). Social relationships of immigrant and resident female mountain gorillas. I. Male_female relationships. American journal of primatology, 28(3), 159-181.
Gorilla gorilla	strict male dominance	0	65	35	NA	Stokes, E. J. (2004). Within_group social relationships among females and adult males in wild western lowland gorillas (Gorilla gorilla gorilla). American Journal of Primatology: Official Journal of the American Society of Primatologists, 64(2), 233-246.
Hapalemur alaotrensis	strict female dominance	86	NA	NA	NA	Waeber&Hemelrijk 2003
Hapalemur griseus	strict female dominance	81	45	51	4	Digby & Stevens 2007
Hapalemur meridionalis	strict female dominance	83	NA	NA	NA	Eppely-etal-2017

Indri indri	strict female dominance	100	NA	NA	NA	Pollock, J. I. (1979). Female dominance in Indri indri. Folia Primatologica, 31(1-2), 143-164.
Indri indri	strict female dominance	100	NA	NA	NA	Pollock, J. I. (1979). Female dominance in Indri indri. Folia Primatologica, 31(1-2), 143-164.
Lagothrix lagotricha	no strict sex bias in dominance	NA	NA	NA	NA	Kavanagh, M., & Dresdale, L. (1975). Observations on the woolly monkey (Lagothrix lagothricha) in northern Colombia. Primates, 16(3), 285-294.
Lagothrix lagotricha	strict male dominance	NA	NA	NA	NA	Stevenson, P. R., Zárate, D. A., Ramírez, M. A., & Henao-Díaz, F. (2015). Social interactions and proximal spacing in woolly monkeys: lonely females looking for male friends. In Dispersing Primate Females (pp. 45-71). Springer, Tokyo.
Lagothrix poeppigii	strict male dominance	0	38	52	10	Di Fiore, A., & Fleischer, R. C. (2005). Social behavior, reproductive strategies, and population genetic structure of Lagothrix poeppigii. International Journal of Primatology, 26(5), 1137-1173.
Lemur catta	strict female dominance	100	11	25	64	Pereira & Kappeler 1996
Lemur catta	strict female dominance	97	43	48	9	Sauther 1993

Lemur catta	strict female dominance	100	NA	NA	NA	Nakamichi & Koyama 1997
Lemur catta	strict female dominance	NA	19	17	64	Pereira, M. E., & Kappeler, P. M. (1997). Divergent systems of agonistic behaviour in lemurid primates. Behaviour, 134(3-4), 225-274.
Lemur catta	strict female dominance	NA	32	38	30	Pereira, M. E., & Kappeler, P. M. (1997). Divergent systems of agonistic behaviour in lemurid primates. Behaviour, 134(3-4), 225-274.
Leontocebus fuscicollis	no strict sex bias in dominance	36	NA	NA	NA	Goldizen, A. W. (1989). Social relationships in a cooperatively polyandrous group of tamarins (Saguinus fuscicollis). Behavioral Ecology and Sociobiology, 24(2), 79-89.
Leontocebus tripartitus	strict female dominance	100	50	NA	50	Kostrup 2003 The social organization and behavior of golden-mantled tamarins, Saguinus tripartitus, in Eastern Ecuador. PhD thesis UC Davis
Leontocebus weddelli	no strict sex bias in dominance	NA	NA	NA	NA	Goldizen AW. 1989. Social relationships in a cooperatively polyandrous group of tamarins (Saguinus fuscicollis). Behavioral Ecology and Sociobiology 24:79-89.
Leontopithecus chrysomelas	no strict sex bias in dominance	NA	NA	NA	NA	Inglett, B. J., French, J. A., Simmons, L. G., & Vires, K. W. (1989). Dynamics of intrafamily aggression and social reintegration in lion tamarins. Zoo Biology, 8(1), 67-78.

Leontopithecus rosalia	no strict sex bias in dominance	NA	NA	NA	NA	Snyder, P. A. (1974). Behavior of Leontopithecus rosalia (golden-lion marmoset) and related species: a review. Journal of Human Evolution, 3(2), 109-122.
Leontopithecus rosalia	no strict sex bias in dominance	NA	NA	NA	NA	Inglett, B. J., French, J. A., Simmons, L. G., & Vires, K. W. (1989). Dynamics of intrafamily aggression and social reintegration in lion tamarins. Zoo Biology, 8(1), 67-78.
Leontopithecus rosalia	no strict sex bias in dominance	67	69	31	0	Inglett, B. J., French, J. A., Simmons, L. G., & Vires, K. W. (1989). Dynamics of intrafamily aggression and social reintegration in lion tamarins. Zoo Biology, 8(1), 67-78.
Lepilemur edwardsi	strict female dominance	97	NA	NA	NA	Rasoloharijaona, S., Randrianambinina, B., & Radespiel, U. (2024). Evidence for female dominance in the Milne_Edwards' sportive lemur (Lepilemur edwardsi). American journal of primatology, e23658.
Lepilemur leucopus	strict female dominance	100	NA	NA	NA	Dröscher & Kappeler 2014
Lepilemur ruficaudatus	no strict sex bias in dominance	50	NA	NA	NA	Hilgartner et al. 2012
Lophocebus ougandae	no strict sex bias in dominance	18	NA	NA	NA	Chalmers, N. (1968). THE SOCIAL BEHAVIOUR OF FREE LIVING MANGABEYS IN UGANDA.

Loris lydekkerianus	strict female dominance	100	60	13	27	Radakrishna & Singh 2002
Macaca arctoides	strict male dominance	9	37	63	0	Rhine RJ (1973) Variation and consistency in the social behavior of two groups of Stumptail macaques (Macaca arctoides). Primates: 14: 21-35.
Macaca arctoides	no strict sex bias in dominance	NA	NA	NA	NA	Butovskaya, M. (1993). Kinship and Different Dominance Styles in Groups of Three Species of the Genus Macaca (M. arctoides, M. mulatta, M. fascicularis). Folia Primatologica, 60(4), 210–224. doi:10.1159/000156694
Macaca assamensis	strict male dominance	NA	NA	NA	NA	Bernstein IS, Cooper MA (1999) Dominance in Assamese macaques. Am. J. Primatol. 28: 283-289
Macaca assamensis	no strict sex bias in dominance	NA	36	24	40	Cooper MA, Bernstein IS, Hemelrijk CK (2005) Reconciliation andrelationship quality in Assamese macaques (Macaca assamensis). AmericanJournal of Primatology 65: 269–282
Macaca cyclopis	no strict sex bias in dominance	NA	NA	NA	NA	Hsu, M. J., Lin, J.-F., & Agoramoorthy, G. (2017). Social implications of fission in wild Formosan macaques at Mount Longevity, Taiwan. Primates, 58(2), 323–334.
Macaca fascicularis	strict male dominance	NA	NA	NA	NA	ANGST, W. (1975). Basic Data and Concepts on the Social Organization of Macaca fascicularis. Primate Behavior, 325–388.doi:10.1016/b978-0-12-534004-5.50011-7

Macaca fascicularis	strict male dominance	NA	NA	NA	NA	ANGST, W. (1975). Basic Data and Concepts on the Social Organization of Macaca fascicularis. Primate Behavior, 325–388.doi:10.1016/b978-0-12-534004-5.50011-7
Macaca fascicularis	no strict sex bias in dominance	19	60	18	22	de Waal, FDM. (1977). The Organization of Agonistic Relations within two Captive Groups of Java_monkeys (Macaca fascicularis). Zeitschrift für Tierpsychologie 44: 225-282
Macaca fascicularis	strict male dominance	3	24	74	2	de Waal, FDM. (1977). The Organization of Agonistic Relations within two Captive Groups of Java_monkeys (Macaca fascicularis). Zeitschrift für Tierpsychologie 44: 225-282
Macaca fascicularis	no strict sex bias in dominance	NA	NA	NA	NA	Butovskaya, M. (1993). Kinship and Different Dominance Styles in Groups of Three Species of the Genus Macaca (M. arctoides, M. mulatta, M. fascicularis). Folia Primatologica, 60(4), 210–224. doi:10.1159/000156694
Macaca fuscata	strict male dominance	2	89	11	0	Mehlman PT, Chapais B (1988) Differential effects of kinship, dominance and the mating season on female allogrooming in a captive group of Macaca fuscata.Primates 29: 195–217
Macaca fuscata	no strict sex bias in dominance	NA	NA	NA	NA	Singh, M., D'Souza, L., & Singh, M. (1992). Hierarchy, kinship and social interaction among Japanese monkeys (Macaca fuscata). Journal of Biosciences, 17(1), 15–27. doi:10.1007/bf02716769
Macaca fuscata	strict male dominance	0	55	36	9	Zhang P, Watanabe K (2014) Intraspecies variation in dominance style in Macaca fuscata, Primates: 55: 69-79

Macaca fuscata	strict male dominance	0	26	68	6	Zhang P, Watanabe K (2014) Intraspecies variation in dominance style in Macaca fuscata, Primates: 55: 69-79
Macaca fuscata	strict male dominance	0	50	50	0	Zhang P, Watanabe K (2014) Intraspecies variation in dominance style in Macaca fuscata, Primates: 55: 69-79
Macaca maura	strict male dominance	0	NA	NA	NA	Petit, O., Thierry, B. (1992) Affiliative function of the silent bared-teeth display in moor macaques (Macaca maurus): Further evidence for the particular status of sulawesi macaques. International Journal of Primatology
Macaca mulatta	strict male dominance	NA	NA	NA	NA	Sueur, C., Petit, O. Organization of Group Members at Departure Is Driven by Social Structure in Macaca . Int J Primatol 29, 1085–1098 (2008). https://doi.org/10.1007/s10764-008-9262-9
Macaca mulatta	strict male dominance	4	29	67	4	De Waal, F. B. M., & Luttrell, L. M. (1985). The formal hierarchy of rhesus macaques: An investigation of the bared-teeth display. American Journal of Primatology, 9(2), 73–85.doi:10.1002/ajp.1350090202
Macaca mulatta	no strict sex bias in dominance	NA	NA	NA	NA	Girod P, Hemelrijk CK (2002) The connection between spatial structure,dominance, aggression and grooming in three species of macaques. In:Hemelrijk CK, ed. Self-organisation and evolution of social behaviour. MonteVerita`, Switzerland: Centro Stefano Franscini. pp 115–116
Macaca mulatta	no strict sex bias in dominance	NA	NA	NA	NA	Ehardt C & Bernstein I (1986) Matrilineal overthrows in Rhesus Monkey groups 7: 157-181

Macaca mulatta	no strict sex bias in dominance	NA	NA	NA	NA	Butovskaya, M. (1993). Kinship and Different Dominance Styles in Groups of Three Species of the Genus Macaca (M. arctoides, M. mulatta, M. fascicularis). Folia Primatologica, 60(4), 210–224.doi:10.1159/000156694
Macaca nemestrina	no strict sex bias in dominance	12	39	54	7	Oi T (1999) Patterns of Dominance and Affiliation in Wild Pig-Tailed Macaques (Macaca nemestrina nemestrina) in West Sumatra, Int. J. Primatol. 11: 339-356
Macaca nigra	strict male dominance	1	17	69	14	Kappeler P.M., Huchard E., Baniel A., Canteloup C., Charpentier M.J.E., Cheng L., Davidian E., Duboscq J., Fichtel C., Hemelrijk C.K., Hoener O.P., Koren L., Micheletta J., Prox L., Saccà T., Seex L., Smit N., Surbeck M., van de Waal E., Girard-Buttoz C. (2022) Sex and dominance: How to assess and interpret intersexual dominance relationships in mammalian societies, Frontiers in Ecology and Evolution 10, 10.3389/fevo.2022.918773. (Table S4, Index 5)
Macaca nigra	strict male dominance	5	23	58	19	Kappeler P.M., Huchard E., Baniel A., Canteloup C., Charpentier M.J.E., Cheng L., Davidian E., Duboscq J., Fichtel C., Hemelrijk C.K., Hoener O.P., Koren L., Micheletta J., Prox L., Saccà T., Seex L., Smit N., Surbeck M., van de Waal E., Girard-Buttoz C. (2022) Sex and dominance: How to assess and interpret intersexual dominance relationships in mammalian societies, Frontiers in Ecology and Evolution 10, 10.3389/fevo.2022.918773. (Table S4, Index 5)
Macaca radiata	no strict sex bias in dominance	18	NA	NA	NA	Sugiyama, Y (1971) Characteristics of social life of Bonnet macaques. Primates, 12: 247-266
Macaca radiata	no strict sex bias in dominance	NA	NA	NA	NA	Koyama, N (1973) Dominance, grooming and clasped-sleeping relationships amonb Bonnet monkeys in India

Macaca radiata	strict male dominance	NA	NA	NA	NA	Boccia, M.L., Laudenslager, M., Reite, M. (1988) Food distribution, dominance and aggressive behaviors in Bonnet macaques, Am J Primatol 16: 123-130.
Macaca silenus	strict male dominance	NA	NA	NA	NA	Newman, R. McKeown, S., Power, T., Quirke, T., O'Riordan R.M. (2020) Introducing three new males into a captive lion-tailed macaque (Macaca silenus) group. JZAR 8: doi.org/10.19227/jzar.v813.396
Macaca sinica	strict male dominance	2	42	44	14	Dittus, W. P. J. (1977). The Social Regulation of Population Density and Age-Sex Distribution in the Toque Monkey. Behaviour, 63, 281–322.
Macaca sylvanus	strict male dominance	0	NA	NA	NA	Kuester, J., & Paul, A. (1996). Female-Female Competition and Male Mate Choice in Barbary Macaques (Macaca Sylvanus). Behaviour, 133(9), 763–790. doi:10.1163/156853996x00468
Macaca sylvanus	strict male dominance	NA	NA	NA	NA	McFarland R & Majolo B 2013 Coping with the cold: predictors of survival in wild Barbary macaques, Macaca sylvanus Biol. Lett. 9: 20130428.
Macaca sylvanus	strict male dominance	NA	NA	NA	NA	McFarland R & Majolo B 2013 Coping with the cold: predictors of survival in wild Barbary macaques, Macaca sylvanus Biol. Lett. 9: 20130428.
Macaca sylvanus	strict male dominance	9	47	50	3	McRoberts, MH. (1970) The social organization of Barbary apes (Macaca sylvana) on Gibraltar, AJPA 33: 83-99.

Macaca sylvanus	strict male dominance	NA	NA	NA	NA	White, D., & Hosey, G. R. (1981). Social organization in captive barbary macaques (Macaca sylvana). Primates, 22(4), 487–493. doi:10.1007/bf02381240
Macaca sylvanus	strict male dominance	NA	NA	NA	NA	Deag, J. M. (1977). Aggression and submission in monkey societies. Animal Behaviour, 25, 465–474. doi:10.1016/0003-3472(77)90021-5
Macaca sylvanus	strict male dominance	NA	NA	NA	NA	Seltmann, A., Majolo, B., Schülke, O., & Ostner, J. (2013). The organization of collective group movements in wild Barbary macaques (Macaca sylvanus): social structure drives processes of group coordination in macaques. PLoS One, 8(6), e67285.
Macaca thibetana	strict male dominance	6	32	56	12	Deng, Z., & Zhao, Q. (1987). Social Structure in a Wild Group of Macaca thibetana at Mount Emei, China. Folia Primatologica, 49(1), 1–10. doi:10.1159/000156304
Macaca thibetana	strict male dominance	6	41	43	16	Berman, C.M., Ionica, C.S. & Li, J. Dominance Style Among Macaca thibetana on Mt. Huangshan, China. International Journal of Primatology 25, 1283–1312 (2004). https://doi.org/10.1023/B:IJOP.0000043963.77801.c3
Macaca tonkeana	no strict sex bias in dominance	NA	NA	NA	NA	Sueur, C., Petit, O. Organization of Group Members at Departure Is Driven by Social Structure in Macaca . Int J Primatol 29, 1085–1098 (2008). https://doi.org/10.1007/s10764-008-9262-9
Macaca tonkeana	strict male dominance	NA	NA	NA	NA	Thierry B, Gauthier C, Peignot P (1990) Social grooming in Tonkean macaques (Macaca tonkeana). International Journal of primatology 11: 357,Äì375.

Macaca tonkeana	no strict sex bias in dominance	NA	NA	NA	NA	Ballesta S., Sadoughi B., Miss F., Whitehouse .J, Aguenounon G., Meunier H. (2021) Assessing the reliability of an automated method for measuring dominance hierarchy in non-human primates. Primates 62:595-607.
Mandrillus sphinx	strict male dominance	NA	NA	NA	NA	Setchell, J. M., Knapp, L. A., & Wickings, E. J. (2006). Violent coalitionary attack by female mandrills against an injured alpha male. American Journal of Primatology, 68(4), 411–418. doi:10.1002/ajp.20234
Mandrillus sphinx	strict male dominance	2	35	60	5	Kappeler P.M., Huchard E., Baniel A., Canteloup C., Charpentier M.J.E., Cheng L., Davidian E., Duboscq J., Fichtel C., Hemelrijk C.K., Hoener O.P., Koren L., Micheletta J., Prox L., Saccà T., Seex L., Smit N., Surbeck M., van de Waal E., Girard-Buttoz C. (2022) Sex and dominance: How to assess and interpret intersexual dominance relationships in mammalian societies, Frontiers in Ecology and Evolution 10, 10.3389/fevo.2022.918773. (Table S4, Index 5)
Microcebus bongolavensis	no strict sex bias in dominance	80	NA	NA	NA	Evasoa, M. R., Zimmermann, E., Hasiniaina, A. F., Rasoloharijaona, S., Randrianambinina, B., & Radespiel, U. (2019). Sources of variation in social tolerance in mouse lemurs (Microcebus spp.). BMC ecology, 19(1), 20.
Microcebus danfossi	strict female dominance	91	NA	NA	NA	Evasoa, M. R., Zimmermann, E., Hasiniaina, A. F., Rasoloharijaona, S., Randrianambinina, B., & Radespiel, U. (2019). Sources of variation in social tolerance in mouse lemurs (Microcebus spp.). BMC ecology, 19(1), 20.
Microcebus griseorufus	strict female dominance	100	NA	NA	NA	Génin, F. (2012). Venus in fur: female power in mouse lemurs Microcebus murinus and M. griseorufus. In Leaping Ahead (pp. 121-126). Springer, New York, NY.
Microcebus lehilahytsara	no strict sex bias in dominance	75	NA	NA	NA	Hohenbrink, S., Schaarschmidt, F., Bünemann, K., Gerberding, S., Zimmermann, E., & Radespiel, U. (2016). Female dominance in two basal primates, Microcebus murinus and Microcebus lehilahytsara: variation and determinants. Animal Behaviour, 122, 145-156.

Microcebus lehilahytsara	no strict sex bias in dominance	49	NA	NA	NA	Hohenbrink, S., Schaarschmidt, F., Bünemann, K., Gerberding, S., Zimmermann, E., & Radespiel, U. (2016). Female dominance in two basal primates, Microcebus murinus and Microcebus lehilahytsara: variation and determinants. Animal Behaviour, 122, 145-156.
Microcebus mamiratra	strict female dominance	97	NA	NA	NA	Evasoa, M. R., Zimmermann, E., Hasiniaina, A. F., Rasoloharijaona, S., Randrianambinina, B., & Radespiel, U. (2019). Sources of variation in social tolerance in mouse lemurs (Microcebus spp.). BMC ecology, 19(1), 20.
Microcebus margotmarshae	strict female dominance	88	NA	NA	NA	Evasoa, M. R., Zimmermann, E., Hasiniaina, A. F., Rasoloharijaona, S., Randrianambinina, B., & Radespiel, U. (2019). Sources of variation in social tolerance in mouse lemurs (Microcebus spp.). BMC ecology, 19(1), 20.
Microcebus murinus	no strict sex bias in dominance	75	NA	NA	NA	Hohenbrink, S., Schaarschmidt, F., Bünemann, K., Gerberding, S., Zimmermann, E., & Radespiel, U. (2016). Female dominance in two basal primates, Microcebus murinus and Microcebus lehilahytsara: variation and determinants. Animal Behaviour, 122, 145-156.
Microcebus murinus	strict female dominance	100	NA	NA	NA	Génin, F. (2012). Venus in fur: female power in mouse lemurs Microcebus murinus and M. griseorufus. In Leaping Ahead (pp. 121-126). Springer, New York, NY.
Microcebus myoxinus	no strict sex bias in dominance	60	NA	NA	NA	Evasoa, M. R., Zimmermann, E., Hasiniaina, A. F., Rasoloharijaona, S., Randrianambinina, B., & Radespiel, U. (2019). Sources of variation in social tolerance in mouse lemurs (Microcebus spp.). BMC ecology, 19(1), 20.
Microcebus ravelobensis	no strict sex bias in dominance	84	NA	NA	NA	Evasoa, M. R., Zimmermann, E., Hasiniaina, A. F., Rasoloharijaona, S., Randrianambinina, B., & Radespiel, U. (2019). Sources of variation in social tolerance in mouse lemurs (Microcebus spp.). BMC ecology, 19(1), 20.

Microcebus ravelobensis	no strict sex bias in dominance	48	NA	NA	NA	Eichmueller, P., Thorén, S., & Radespiel, U. (2013). The lack of female dominance in golden_brown mouse lemurs suggests alternative routes in lemur social evolution. American Journal of Physical Anthropology, 150(1), 158-164.
Miopithecus talapoin	no strict sex bias in dominance	NA	NA	NA	NA	Dixson A.F., Scruton D. M., Herbert J. (1975). Behaviour of the Talapoin monkey (Miopithecus talapoin) studied in groups, in the laboratory. American Journal of Physical Anthropology 176(2): 177-210. doi:10.1111/j.1469-7998.1975.tb03192.x
Miopithecus talapoin	no strict sex bias in dominance	NA	NA	NA	NA	Dixson A.F., Scruton D. M., Herbert J. (1975). Behaviour of the Talapoin monkey (Miopithecus talapoin) studied in groups, in the laboratory. American Journal of Physical Anthropology 176(2): 177-210. doi:10.1111/j.1469-7998.1975.tb03192.x
Miopithecus talapoin	strict female dominance	NA	NA	NA	NA	Dixson A.F., Scruton D. M., Herbert J. (1975). Behaviour of the Talapoin monkey (Miopithecus talapoin) studied in groups, in the laboratory. American Journal of Physical Anthropology 176(2): 177-210. doi:10.1111/j.1469-7998.1975.tb03192.x
Miopithecus talapoin	strict female dominance	NA	NA	NA	NA	Dixson A.F., Scruton D. M., Herbert J. (1975). Behaviour of the Talapoin monkey (Miopithecus talapoin) studied in groups, in the laboratory. American Journal of Physical Anthropology 176(2): 177-210. doi:10.1111/j.1469-7998.1975.tb03192.x
Miopithecus talapoin	no strict sex bias in dominance	73	57	33	10	Wolfheim, J. H. (1977). A Quantitative Analysis of the Organization of a Group of Captive Talapoin Monkeys (Miopithecus talapoin). Folia Primatologica, 27(1), 1–27.
Miopithecus talapoin	strict male dominance	NA	NA	NA	NA	Keverne, E. B., Leonard, R. A., Scruton, D. M., & Young, S. K. (1978). Visual monitoring in social groups of talapoin monkeys (Miopithecus talapoin). Animal Behaviour, 26, 933–944. doi:10.1016/0003-3472(78)90157-4

Miopithecus talapoin	no strict sex bias in dominance	NA	NA	NA	NA	Keverne, E. B., Leonard, R. A., Scruton, D. M., & Young, S. K. (1978). Visual monitoring in social groups of talapoin monkeys (Miopithecus talapoin). Animal Behaviour, 26, 933–944. doi:10.1016/0003-3472(78)90157-4
Miopithecus talapoin	strict female dominance	NA	NA	NA	NA	Keverne, E. B., Leonard, R. A., Scruton, D. M., & Young, S. K. (1978). Visual monitoring in social groups of talapoin monkeys (Miopithecus talapoin). Animal Behaviour, 26, 933–944. doi:10.1016/0003-3472(78)90157-4
Miopithecus talapoin	no strict sex bias in dominance	NA	NA	NA	NA	Keverne, E. B., Leonard, R. A., Scruton, D. M., & Young, S. K. (1978). Visual monitoring in social groups of talapoin monkeys (Miopithecus talapoin). Animal Behaviour, 26, 933–944. doi:10.1016/0003-3472(78)90157-4
Nasalis larvatus	strict male dominance	0	NA	NA	NA	Yeager, C. P. (1990). Proboscis monkey (Nasalis larvatus) social organization: Group structure. American Journal of Primatology, 20(2), 95–106.doi:10.1002/ajp.1350200204
Pan paniscus	no strict sex bias in dominance	48	31	16	53	Furuichi, T. (1997). Agonistic interactions and matrifocal dominance rank of wild bonobos (Pan paniscus) at Wamba. International Journal of Primatology, 18(6), 855-875.
Pan paniscus	no strict sex bias in dominance	70	38	23	39	Fruth, B., & Hohmann, G. (2003). Intra-and inter-sexual aggression by bonobos in the context of mating. Behaviour, 140(11-12), 1389-1413.
Pan paniscus	no strict sex bias in dominance	62	NA	NA	NA	Surbeck, M., & Hohmann, G. (2013). Intersexual dominance relationships and the influence of leverage on the outcome of conflicts in wild bonobos (Pan paniscus). Behavioral Ecology and Sociobiology, 67(11), 1767-1780.

Pan paniscus	no strict sex bias in dominance	45	31	7	62	Kappeler P.M., Huchard E., Baniel A., Canteloup C., Charpentier M.J.E., Cheng L., Davidian E., Duboscq J., Fichtel C., Hemelrijk C.K., Hoener O.P., Koren L., Micheletta J., Prox L., Saccà T., Seex L., Smit N., Surbeck M., van de Waal E., Girard-Buttoz C. (2022) Sex and dominance: How to assess and interpret intersexual dominance relationships in mammalian societies, Frontiers in Ecology and Evolution 10, 10.3389/fevo.2022.918773. (Table S4, Index 5)
Pan paniscus	no strict sex bias in dominance	79	44	9	47	Kappeler P.M., Huchard E., Baniel A., Canteloup C., Charpentier M.J.E., Cheng L., Davidian E., Duboscq J., Fichtel C., Hemelrijk C.K., Hoener O.P., Koren L., Micheletta J., Prox L., Saccà T., Seex L., Smit N., Surbeck M., van de Waal E., Girard-Buttoz C. (2022) Sex and dominance: How to assess and interpret intersexual dominance relationships in mammalian societies, Frontiers in Ecology and Evolution 10, 10.3389/fevo.2022.918773. (Table S4, Index 5)
Pan troglodytes	strict male dominance	5	34	0	66	Muller, M. N. (2002). Agonistic relations among Kanyawara chimpanzees. Behavioural diversity in chimpanzees and bonobos, 112-124.
Pan troglodytes	strict male dominance	4	50	16	34	Muller, M. N. (2002). Agonistic relations among Kanyawara chimpanzees. Behavioural diversity in chimpanzees and bonobos, 112-124.
Pan troglodytes	strict male dominance	NA	NA	NA	NA	Stumpf, R. M., & Boesch, C. (2010). Male aggression and sexual coercion in wild West African chimpanzees, Pan troglodytes verus. Animal Behaviour, 79(2), 333-342.
Papio anubis	strict male dominance	3	NA	NA	NA	Harding, R. S. O. (1980). Agonism, ranking, and the social behavior of adult male baboons. American Journal of Physical Anthropology, 53(2), 203–216. doi:10.1002/ajpa.1330530205

Papio anubis	strict male dominance	NA	NA	NA	NA	Smuts, B. (1985) Sex and friendships in baboons, Harvard University Press, 1999.
Papio cynocephalus	strict male dominance	NA	NA	NA	NA	Post, D. G., Hausfater, G., & McCuskey, S. (1980). Feeding Behavior of Yellow Baboons (Papio cynocephalus): Relationship to Age, Gender and Dominance Rank. Folia Primatologica, 34(3-4), 170–195.doi:10.1159/000155954
Papio cynocephalus	strict male dominance	9	26	6	68	Collins, D. A. (1981). Social behaviour and patterns of mating among adult yellow baboons (Papio c. cynocephalus. L. 1766).
Papio hamadryas	strict male dominance	0	NA	NA	NA	Polo, P., & Colmenares, F. (2012). Behavioural processes in social context: Female abductions, male herding and female grooming in hamadryas baboons. Behavioural Processes, 90(2), 238–245.doi:10.1016/j.beproc.2012.02.004
Papio hamadryas	strict male dominance	0	NA	NA	NA	Swedell, L., & Schreier, A. (2009). Male aggression towards females in hamadryas baboons: conditioning, coercion, and control. Sexual coercion in primates and humans: an evolutionary perspective on male aggression against females. Harvard University Press, Cambridge, 244-268.
Papio hamadryas	strict male dominance	NA	NA	NA	NA	Kummer, H. 1968. Social Organization of Hamadryas Baboons. A Field Study. Chicago & London: Chicago University Press.
Papio papio	no strict sex bias in dominance	15	NA	NA	NA	Kalbitzer, U., Heistermann, M., Cheney, D., Seyfarth, R., & Fischer, J. (2015). Social behavior and patterns of testosterone and glucocorticoid levels differ between male chacma and Guinea baboons. Hormones and behavior, 75, 100-110.

Papio papio	no strict sex bias in dominance	20	NA	NA	NA	Goffe, A.S., Zinner, D., Fischer J. (2016) Sex and friendship in a multilevel society: behavioural patterns and associations between female and male Guinea baboons. Behav. Ecol. Sociobiol. 70:323-336.
Papio ursinus	no strict sex bias in dominance	NA	NA	NA	NA	Kalbitzer, U., Heistermann, M., Cheney, D., Seyfarth, R., & Fischer, J. (2015). Social behavior and patterns of testosterone and glucocorticoid levels differ between male chacma and Guinea baboons. Hormones and behavior, 75, 100-110.
Papio ursinus	strict male dominance	NA	NA	NA	NA	Kitchen, Dawn M., et al. "The causes and consequences of male aggression directed at female chacma baboons." Sexual Coercion in Primates and Humans: An Evolutionary Perspective on Male Aggression Against Females (2009): 128-156.
Papio ursinus	strict male dominance	NA	NA	NA	NA	Baniel, A., Cowlishaw, G., & Huchard, E. (2017). Male violence and sexual intimidation in a wild primate society. Current Biology, 27(14), 2163-2168.
Papio ursinus	strict male dominance	1	28	59	13	Kappeler P.M., Huchard E., Baniel A., Canteloup C., Charpentier M.J.E., Cheng L., Davidian E., Duboscq J., Fichtel C., Hemelrijk C.K., Hoener O.P., Koren L., Micheletta J., Prox L., Saccà T., Seex L., Smit N., Surbeck M., van de Waal E., Girard-Buttoz C. (2022) Sex and dominance: How to assess and interpret intersexual dominance relationships in mammalian societies, Frontiers in Ecology and Evolution 10, 10.3389/fevo.2022.918773. (Table S4, Index 5)
Papio ursinus	strict male dominance	1	45	46	9	Kappeler P.M., Huchard E., Baniel A., Canteloup C., Charpentier M.J.E., Cheng L., Davidian E., Duboscq J., Fichtel C., Hemelrijk C.K., Hoener O.P., Koren L., Micheletta J., Prox L., Saccà T., Seex L., Smit N., Surbeck M., van de Waal E., Girard-Buttoz C. (2022) Sex and dominance: How to assess and interpret intersexual dominance relationships in mammalian societies, Frontiers in Ecology and Evolution 10, 10.3389/fevo.2022.918773. (Table S4, Index 5)

Papio ursinus	strict male dominance	3	46	36	18	Kappeler P.M., Huchard E., Baniel A., Canteloup C., Charpentier M.J.E., Cheng L., Davidian E., Duboscq J., Fichtel C., Hemelrijk C.K., Hoener O.P., Koren L., Micheletta J., Prox L., Saccà T., Seex L., Smit N., Surbeck M., van de Waal E., Girard-Buttoz C. (2022) Sex and dominance: How to assess and interpret intersexual dominance relationships in mammalian societies, Frontiers in Ecology and Evolution 10, 10.3389/fevo.2022.918773. (Table S4, Index 5)
Papio ursinus	strict male dominance	0	46	43	11	Kappeler P.M., Huchard E., Baniel A., Canteloup C., Charpentier M.J.E., Cheng L., Davidian E., Duboscq J., Fichtel C., Hemelrijk C.K., Hoener O.P., Koren L., Micheletta J., Prox L., Saccà T., Seex L., Smit N., Surbeck M., van de Waal E., Girard-Buttoz C. (2022) Sex and dominance: How to assess and interpret intersexual dominance relationships in mammalian societies, Frontiers in Ecology and Evolution 10, 10.3389/fevo.2022.918773. (Table S4, Index 5)
Perodicticus edwardsi	no strict sex bias in dominance	NA	NA	NA	NA	Pimnley et al. 2005
Phaner pallescens	strict female dominance	100	NA	NA	NA	Schülke&Kappeler 2003
Pithecia aequatorialis	no strict sex bias in dominance	NA	NA	NA	NA	Porter, A. M., Grote, M. N., Isbell, L. A., Fernandez-Duque, E., & Di Fiore, A. (2017). Delayed dispersal and immigration in equatorial sakis (Pithecia aequatorialis): factors in the transition from pair-to group-living. Folia Primatologica, 88(1), 11-27.
Pithecia pithecia	no strict sex bias in dominance	NA	NA	NA	NA	Harrison, A. L., & Norconk, M. A. (1999). Social dominance in a group of white-faced sakis (Pithecia pithecia) in the context of a rare and limited resource. American Journal of Primatology, 49(1), 60.

Presbytis thomasi	strict male dominance	NA	NA	NA	NA	Steenbeek, R., & Sterck, E..M. (1997). Female Dominance Relationships and Food Competition in the Sympatric Thomas Langur and Long-Tailed Macaque, Behaviour, 134(9-10), 749-774. doi: https://doi.org/10.1163/156853997X00052
Presbytis thomasi	strict male dominance	NA	NA	NA	NA	Steenbeek, R., & Sterck, E..M. (1997). Female Dominance Relationships and Food Competition in the Sympatric Thomas Langur and Long-Tailed Macaque, Behaviour, 134(9-10), 749-774. doi: https://doi.org/10.1163/156853997X00052
Presbytis thomasi	strict male dominance	NA	NA	NA	NA	Steenbeek, R., & Sterck, E..M. (1997). Female Dominance Relationships and Food Competition in the Sympatric Thomas Langur and Long-Tailed Macaque, Behaviour, 134(9-10), 749-774. doi: https://doi.org/10.1163/156853997X00052
Propithecus coquereli	strict female dominance	100	NA	NA	NA	Kubzdela, K. S., Richard, A. F., & Pereira, M. E. (1992). Social relations in semi_free_ranging sifakas (Propithecus verreauxi coquereli) and the question of female dominance. American Journal of Primatology, 28(2), 139-145.
Propithecus coquereli	strict female dominance	100	NA	NA	NA	Kubzdela, K. S., Richard, A. F., & Pereira, M. E. (1992). Social relations in semi_free_ranging sifakas (Propithecus verreauxi coquereli) and the question of female dominance. American Journal of Primatology, 28(2), 139-145.
Propithecus coronatus	no strict sex bias in dominance	82	55	25	20	Ramanamisata et al (2014) Social behavior and dominance of the crowned sifaka (Propithecus coronatus) in northwestern Madagascar." Primate Conservation 2014.28 (2014): 93-98.
Propithecus coronatus	no strict sex bias in dominance	80	63	13	24	Ramanamisata et al (2014) Social behavior and dominance of the crowned sifaka (Propithecus coronatus) in northwestern Madagascar." Primate Conservation 2014.28 (2014): 93-98.

Propithecus coronatus	no strict sex bias in dominance	78	82	9	9	Ramanamisata et al (2014) Social behavior and dominance of the crowned sifaka (Propithecus coronatus) in northwestern Madagascar." Primate Conservation 2014.28 (2014): 93-98.
Propithecus diadema	strict female dominance	100	NA	NA	NA	Rasolonjatovo, S. M., & Irwin, M. T. (2020). Exploring Social Dominance in Wild Diademed Sifakas (Propithecus diadema): Females Are Dominant, but It Is Subtle and the Benefits Are Not Clear. Folia Primatologica, 91(4), 385-398.
Propithecus diadema	no strict sex bias in dominance	63	NA	NA	NA	Rasolonjatovo, S. M., & Irwin, M. T. (2020). Exploring Social Dominance in Wild Diademed Sifakas (Propithecus diadema): Females Are Dominant, but It Is Subtle and the Benefits Are Not Clear. Folia Primatologica, 91(4), 385-398.
Propithecus diadema	strict female dominance	100	NA	NA	NA	Rasolonjatovo, S. M., & Irwin, M. T. (2020). Exploring Social Dominance in Wild Diademed Sifakas (Propithecus diadema): Females Are Dominant, but It Is Subtle and the Benefits Are Not Clear. Folia Primatologica, 91(4), 385-398.
Propithecus edwardsi	strict female dominance	100	57	22	21	Pochron, S. T., Fitzgerald, J., Gilbert, C. C., Lawrence, D., Grgas, M., Rakotonirina, G., ... & Wright, P. C. (2003). Patterns of female dominance in Propithecus diadema edwardsi of Ranomafana National Park, Madagascar. American Journal of Primatology: Official Journal of the American Society of Primatologists, 61(4), 173-185.
Propithecus verreauxi	strict female dominance	95	72	11	17	Kappeler, P. M., Mass, V., & Port, M. (2009). (Propithecus verreauxi) in the Kirindy Forest CFPF, Madagascar. American Journal of Physical Anthropology: The Official Publication of the American Association of Physical Anthropologists, 140(3), 487-497.
Propithecus verreauxi	strict female dominance	99	NA	NA	NA	Voyt, R. A., Sandel, A. A., Ortiz, K. M., & Lewis, R. J. (2019). Female power in Verreaux’s sifaka (Propithecus verreauxi) is based on maturity, not body size. International Journal of Primatology, 40(3), 417-434., Leimberger, K. G., & Lewis, R. J. (2017). Patterns of male dispersal in Verreaux's sifaka (Propithecus verreauxi) at Kirindy

						Mitea National Park. American journal of primatology, 79(7), e22455.
Propithecus verreauxi	strict female dominance	NA	NA	NA	NA	Voyt, R. A., Sandel, A. A., Ortiz, K. M., & Lewis, R. J. (2019). Female power in Verreaux’s sifaka (Propithecus verreauxi) is based on maturity, not body size. International Journal of Primatology, 40(3), 417-434., Leimberger, K. G., & Lewis, R. J. (2017). Patterns of male dispersal in Verreaux's sifaka (Propithecus verreauxi) at Kirindy Mitea National Park. American journal of primatology, 79(7), e22455.
Propithecus verreauxi	strict female dominance	99	45	9	46	Voyt, R. A., Sandel, A. A., Ortiz, K. M., & Lewis, R. J. (2019). Female power in Verreaux’s sifaka (Propithecus verreauxi) is based on maturity, not body size. International Journal of Primatology, 40(3), 417-434., Leimberger, K. G., & Lewis, R. J. (2017). Patterns of male dispersal in Verreaux's sifaka (Propithecus verreauxi) at Kirindy Mitea National Park. American journal of primatology, 79(7), e22455.
Propithecus verreauxi	no strict sex bias in dominance	NA	NA	NA	NA	Voyt, R. A., Sandel, A. A., Ortiz, K. M., & Lewis, R. J. (2019). Female power in Verreaux’s sifaka (Propithecus verreauxi) is based on maturity, not body size. International Journal of Primatology, 40(3), 417-434., Leimberger, K. G., & Lewis, R. J. (2017). Patterns of male dispersal in Verreaux's sifaka (Propithecus verreauxi) at Kirindy Mitea National Park. American journal of primatology, 79(7), e22455.
Propithecus verreauxi	strict female dominance	94	42	0	58	Kappeler P.M., Huchard E., Baniel A., Canteloup C., Charpentier M.J.E., Cheng L., Davidian E., Duboscq J., Fichtel C., Hemelrijk C.K., Hoener O.P., Koren L., Micheletta J., Prox L., Saccà T., Seex L., Smit N., Surbeck M., van de Waal E., Girard-Buttoz C. (2022) Sex and dominance: How to assess and interpret intersexual dominance relationships in mammalian societies, Frontiers in Ecology and Evolution 10, 10.3389/fevo.2022.918773. (Table S4, Index 5)

Propithecus verreauxi	strict female dominance	100	79	0	21	Kappeler P.M., Huchard E., Baniel A., Canteloup C., Charpentier M.J.E., Cheng L., Davidian E., Duboscq J., Fichtel C., Hemelrijk C.K., Hoener O.P., Koren L., Micheletta J., Prox L., Saccà T., Seex L., Smit N., Surbeck M., van de Waal E., Girard-Buttoz C. (2022) Sex and dominance: How to assess and interpret intersexual dominance relationships in mammalian societies, Frontiers in Ecology and Evolution 10, 10.3389/fevo.2022.918773. (Table S4, Index 5)
Propithecus verreauxi	strict female dominance	100	85	0	15	Kappeler P.M., Huchard E., Baniel A., Canteloup C., Charpentier M.J.E., Cheng L., Davidian E., Duboscq J., Fichtel C., Hemelrijk C.K., Hoener O.P., Koren L., Micheletta J., Prox L., Saccà T., Seex L., Smit N., Surbeck M., van de Waal E., Girard-Buttoz C. (2022) Sex and dominance: How to assess and interpret intersexual dominance relationships in mammalian societies, Frontiers in Ecology and Evolution 10, 10.3389/fevo.2022.918773. (Table S4, Index 5)
Propithecus verreauxi	strict female dominance	96	NA	NA	NA	Lewis R. J., Bueno G. L., Di Fiore A. (2022) Variation in Female Leverage: The Influence of Kinship and Market Effects on the Extent of Female Power Over Males in Verreaux's Sifaka, Frontiers in Ecology and Evolution 10, 10.3389/fevo.2022.851880
Pygathrix nemaeus	strict male dominance	NA	NA	NA	NA	Kavanagh M (1978) The social behaviour of doucs (Pygathrix nemaeus) at San Diego Zoo
Rhinopithecus bieti	strict male dominance	NA	NA	NA	NA	Cui et al (2014) Dominance hierarchy and social relationships in a group of Captive black-and-white snub-nosed monkeys (Rhinopithecus bieti)
Saguinus bicolor	no strict sex bias in dominance	NA	NA	NA	NA	Scotson, L. Developing a Behavioural Measure of Stress in Captive Pied Tamarins Saguinus bicolor. Bsc Thesis Aberdeen 2006

Saguinus imperator	no strict sex bias in dominance	38	NA	NA	NA	Knox, K. L., & Sade, D. S. (1991). Social behavior of the emperor tamarin in captivity: components of agonistic display and the agonistic network. International journal of primatology, 12(5), 439-480.
Saguinus imperator	no strict sex bias in dominance	16	59	31	10	Knox, K. L., & Sade, D. S. (1991). Social behavior of the emperor tamarin in captivity: components of agonistic display and the agonistic network. International journal of primatology, 12(5), 439-480.
Saguinus mystax	no strict sex bias in dominance	NA	NA	NA	NA	Heymann, E. W. (1990). Social behaviour and infant carrying in a group of moustached tamarins, Saguinus mystax (Primates: Platyrrhini: Callitrichidae), on Padre Isla, Peruvian Amazonia. Primates, 31(2), 183-196.
Saguinus mystax	no strict sex bias in dominance	33	NA	NA	NA	Huck, M., Löttker, P., & Heymann, E. W. (2004). Proximate mechanisms of reproductive monopolization in male moustached tamarins (Saguinus mystax). American Journal of Primatology: Official Journal of the American Society of Primatologists, 64(1), 39-56.
Saguinus mystax	no strict sex bias in dominance	50	NA	NA	NA	Huck, M., Löttker, P., & Heymann, E. W. (2004). Proximate mechanisms of reproductive monopolization in male moustached tamarins (Saguinus mystax). American Journal of Primatology: Official Journal of the American Society of Primatologists, 64(1), 39-56.
Saguinus mystax	strict male dominance	0	NA	NA	NA	Heymann, E. W. (1996). Social behavior of wild moustached tamarins, Saguinus mystax, at the Estación Biológica Quebrada Blanco, Peruvian Amazonia. American Journal of Primatology, 38(1), 101-113.
Saguinus mystax	strict male dominance	0	71	5	24	Heymann, E. W. (1996). Social behavior of wild moustached tamarins, Saguinus mystax, at the Estación Biológica Quebrada Blanco, Peruvian Amazonia. American Journal of Primatology, 38(1), 101-113.

Saimiri boliviensis	strict male dominance	9	NA	NA	NA	Boinski, S., Sughrue, K., Selvaggi, L., Quatrone, R., Henry, M., & Cropp, S. (2002). An expanded test of the ecological model of primate social evolution: competitive regimes and female bonding in three species of squirrel monkeys (Saimiri oerstedii, S. boliviensis and S. sciureus). Behaviour, 139(2), 227-261.
Saimiri collinsi	no strict sex bias in dominance	NA	NA	NA	NA	Pinheiro, T., & Lopes, M. A. (2018). Hierarchical structure and the influence of individual attributes in the captive squirrel monkey (Saimiri collinsi). Primates, 59(5), 475-482.
Saimiri oerstedii	no strict sex bias in dominance	NA	NA	NA	NA	Boinski, S., Sughrue, K., Selvaggi, L., Quatrone, R., Henry, M., & Cropp, S. (2002). An expanded test of the ecological model of primate social evolution: competitive regimes and female bonding in three species of squirrel monkeys (Saimiri oerstedii, S. boliviensis and S. sciureus). Behaviour, 139(2), 227-261.
Saimiri sciureus	strict male dominance	9	78	22	NA	Boinski, S., Sughrue, K., Selvaggi, L., Quatrone, R., Henry, M., & Cropp, S. (2002). An expanded test of the ecological model of primate social evolution: competitive regimes and female bonding in three species of squirrel monkeys (Saimiri oerstedii, S. boliviensis and S. sciureus). Behaviour, 139(2), 227-261.
Saimiri sciureus	strict female dominance	NA	NA	NA	NA	Baldwin, J. D. (1968). The social behavior of adult male squirrel monkeys (Saimiri sciureus) in a seminatural environment. Folia primatologica, 9(3-4), 281-314.
Sapajus apella	no strict sex bias in dominance	16	27	16	57	Janson, C. (1985). Aggresive competition and individual food consumption in wild brown capuchin monkeys (Cebus apella). Behavioral Ecology and Sociobiology, 18, 125-138.
Sapajus apella	no strict sex bias in dominance	50	39	29	32	Izar, P. (2004). Female social relationships of Cebus apella nigritus in a southeastern Atlantic forest: an analysis through ecological models of primate social evolution. Behaviour, 141(1), 71-99.

Sapajus apella	no strict sex bias in dominance	NA	NA	NA	NA	Janson, C. H. (1990). Ecological consequences of individual spatial choice in foraging groups of brown capuchin monkeys, Cebus apella. Animal Behaviour, 40(5), 922-934.
Sapajus cay	no strict sex bias in dominance	NA	NA	NA	NA	Rímoli, J., Ludwig, G., Lynch-Alfaro, J., Melo, F., Mollinedo, J., & Santos, M. (2018). Sapajus cay. The IUCN Red List of Threatened Species, 2018-2.
Sapajus libidinosus	no strict sex bias in dominance	38	25	39	36	Verderane, M. P., Izar, P., Visalberghi, E., & Fragaszy, D. M. (2013). Socioecology of wild bearded capuchin monkeys (Sapajus libidinosus): an analysis of social relationships among female primates that use tools in feeding. Behaviour, 150(6), 659-689.
Sapajus libidinosus	no strict sex bias in dominance	54	42	28	30	Verderane, M. P., Izar, P., Visalberghi, E., & Fragaszy, D. M. (2013). Socioecology of wild bearded capuchin monkeys (Sapajus libidinosus): an analysis of social relationships among female primates that use tools in feeding. Behaviour, 150(6), 659-689.
Semnopithecus entellus	strict male dominance	10	NA	NA	NA	Koenig A., Miles A., Riaz D., Borries C. (2022) Intersexual Agonism in Gray Langurs Reflects Male Dominance and Feeding Competition, Frontiers in Ecology and Evolution 10, 10.3389/fevo.2022.860437
Semnopithecus schistaceus	strict male dominance	3	NA	NA	NA	Koenig A., Miles A., Riaz D., Borries C. (2022) Intersexual Agonism in Gray Langurs Reflects Male Dominance and Feeding Competition, Frontiers in Ecology and Evolution 10, 10.3389/fevo.2022.860437
Semnopithecus schistaceus	strict male dominance	10	NA	NA	NA	Koenig A., Miles A., Riaz D., Borries C. (2022) Intersexual Agonism in Gray Langurs Reflects Male Dominance and Feeding Competition, Frontiers in Ecology and Evolution 10, 10.3389/fevo.2022.860437

Semnopithecus schistaceus	strict male dominance	0	NA	NA	NA	Koenig A., Miles A., Riaz D., Borries C. (2022) Intersexual Agonism in Gray Langurs Reflects Male Dominance and Feeding Competition, Frontiers in Ecology and Evolution 10, 10.3389/fevo.2022.860437
Semnopithecus schistaceus	strict male dominance	0	NA	NA	NA	Koenig A., Miles A., Riaz D., Borries C. (2022) Intersexual Agonism in Gray Langurs Reflects Male Dominance and Feeding Competition, Frontiers in Ecology and Evolution 10, 10.3389/fevo.2022.860437
Semnopithecus schistaceus	strict male dominance	NA	NA	NA	NA	Koenig A., Miles A., Riaz D., Borries C. (2022) Intersexual Agonism in Gray Langurs Reflects Male Dominance and Feeding Competition, Frontiers in Ecology and Evolution 10, 10.3389/fevo.2022.860437
Semnopithecus schistaceus	strict male dominance	0	NA	NA	NA	Koenig A., Miles A., Riaz D., Borries C. (2022) Intersexual Agonism in Gray Langurs Reflects Male Dominance and Feeding Competition, Frontiers in Ecology and Evolution 10, 10.3389/fevo.2022.860437
Theropithecus gelada	no strict sex bias in dominance	NA	NA	NA	NA	Pallante et al (2016) Pallante, V., Stanyon, R., & Palagi, E. (2016). Agonistic support towards victims buffers aggression in geladas (Theropithecus gelada). Behaviour, 153(9-11), 1217-1243.
Trachypithecus cristatus	strict male dominance	NA	NA	NA	NA	Amarasinghe et al (2009) Social behaviours of captive Trachypithecus cristatus (Mammalia: Cercopithecidae) in the National Zoological Gardens of Sri Lanka
Trachypithecus obscurus	strict male dominance	NA	NA	NA	NA	Arnold, K., & Barton, R. A. (2001). Postconflict behavior of spectacled leaf monkeys (Trachypithecus obscurus). I. Reconciliation. *International Journal of Primatology*, *22*, 243-266.

Trachypithecus obscurus	strict male dominance	NA	NA	NA	NA	Arnold, K., & Barton, R. A. (2001). Postconflict behavior of spectacled leaf monkeys (Trachypithecus obscurus). I. Reconciliation. *International Journal of Primatology*, *22*, 243-266.
Varecia rubra	strict female dominance	100	NA	NA	NA	Meyer, C., Gallo, T., & Schultz, S. T. (1999). Female dominance in captive red ruffed lemurs, Varecia variegata rubra (Primates, Lemuridae). *Folia primatologica*, *70*(6), 358.
Varecia rubra	strict female dominance	94	NA	NA	NA	Raps, S., & White, F. J. (1995). Female social dominance in semi-free-ranging ruffed lemurs (Varecia variegata). *Folia Primatologica*, *65*(3), 163-168.
Varecia rubra	strict female dominance	99	NA	NA	NA	Raps, S., & White, F. J. (1995). Female social dominance in semi-free-ranging ruffed lemurs (Varecia variegata). *Folia Primatologica*, *65*(3), 163-168.
Varecia variegata	strict female dominance	97	NA	NA	NA	Raps, S., & White, F. J. (1995). Female social dominance in semi-free-ranging ruffed lemurs (Varecia variegata). *Folia Primatologica*, *65*(3), 163-168.
Varecia variegata	strict female dominance	100	NA	NA	NA	Kaufman, R. (1991). Female dominance in semifree-ranging black-and-white ruffed lemurs, Varecia variegata variegata. *Folia Primatologica*, *57*(1), 39-41.
Varecia variegata	no strict sex bias in dominance	78	NA	NA	NA	Overdorff, D. J., Erhart, E. M., & Mutschler, T. (2005). Does female dominance facilitate feeding priority in black-and-white ruffed lemurs (Varecia variegata) in southeastern Madagascar?. *American Journal of Primatology: Official Journal of the American Society of Primatologists*, *66*(1), 7-22.

Supplementary File 1: Phylogenetic relationship among the species in our sample (Newick format)

(((Daubentonia_madagascariensis:41.13816293,((((Phaner_pallescens:18.03623594,((Lepilemur_leucopus:4.188967615,Lepilemur_ruficaudatu
s:4.18896766):1.319452764,Lepilemur_edwardsi:5.508420427):12.52781561):1.034798954,((((Microcebus_mamiratra:1.791443443,Microceb
us_margotmarshae:1.791443491):1.37534716,((Microcebus_danfossi:0.9883348525,Microcebus_lehilahytsara:0.988334735):0.9597153401,M
icrocebus_myoxinus:1.948050152):1.218740496):1.427260345,(Microcebus_ravelobensis:0.5388640212,Microcebus_bongolavensis:0.538864
0134):4.055186961):1.217040323,(Microcebus_griseorufus:3.826369322,Microcebus_murinus:3.826369361):1.984721739):13.25994361):2.12
6270899,((Varecia_variegata:0.9203742124,Varecia_rubra:0.9203742124):12.42396876,((Lemur_catta:5.347535666,(Hapalemur_griseus:2.438
312698,(Hapalemur_alaotrensis:0.8000427314,Hapalemur_meridionalis:0.8000426846):1.638269999):2.909223279):4.989256628,((Eulemur_
macaco:1.584937186,Eulemur_flavifrons:1.584937186):2.036241788,(Eulemur_coronatus:3.224545814,(Eulemur_rubriventer:2.704666201,(E
ulemur_rufifrons:0.837709231,Eulemur_fulvus:0.8377092239):1.866956906):0.5198796641):0.3966331355):6.71561344):3.007550608):7.852
962641):1.667831607,(Indri_indri:11.23552531,(((Propithecus_verreauxi:2.149290042,Propithecus_coquereli:2.149290084):2.473202165,(Pro
pithecus_coronatus:1.29247376,(Propithecus_edwardsi:0.8204503624,Propithecus_diadema:0.8204503624):0.4720232479):3.3300185):4.549
524164,Avahi_occidentalis:9.172016371):2.063508974):11.62961206):18.27302362):8.239797704,(Perodicticus_edwardsi:22.10052238,Loris_l
ydekkerianus:22.10052238):27.27744):17.66800421,(((((Pithecia_pithecia:4.23050019,Pithecia_aequatorialis:4.230500164):4.676427997,((Cac
ajao_calvus:2.323523517,(Cacajao_ouakary:1.306646046,Cacajao_melanocephalus:
1.306646046):1.016877):5.282964057,Chiropotes_sagulatus:7.606488):1.300441):5.840985604,(Callicebus_coimbrai:5.504193898,(Callicebus_
moloch:2.108810517,Callicebus_cupreus:2.108810522):3.395383397):9.243719995):2.316632987,((((((Saguinus_imperator:3.229111706,Sagui
nus_mystax:3.229111908):1.968283136,Saguinus_bicolor:5.197394964):1.374896305,((Leontocebus_fuscicollis:1.015255664,Leontocebus_we
ddelli:1.015255666):0.7677378418,Leontocebus_tripartitus:1.782993576):4.789297692):4.356328826,(((Callithrix_jacchus:4.177287305,Cebue

lla_pygmaea:4.177287333):1.036483479,(Callithrix_flaviceps:3.232267948,Callithrix_penicillata:3.232268001):1.981502925):4.954654171,(Leontopithecus_chrysomelas:0.3608203722,Leontopithecus_rosalia:0.3608203722):9.807604719):0.760195194):3.570072013,((((Saimiri_oerstedii:1.092650171,(Saimiri_collinsi:0.123193902,Saimiri_sciureus:0.123193902):0.9694562):0.4111320269,Saimiri_boliviensis:1.50378214):10.75882646,(((Sapajus_apella:0.5197849706,Sapajus_libidinosus:0.5197849803):0.7198968636,Sapajus_cay:1.239681871):2.604837462,((Cebus_albifrons:1.188381801,Cebus_olivaceus:1.188381801):0.4341067483,Cebus_capucinus:1.622488614):2.222030723):8.418089293):1.800609353,Aotus_azarae:14.06321783):0.4354742251):1.520809424,((Alouatta_seniculus:4.717863412,((Alouatta_pigra:1.382893336,Alouatta_palliata:1.382893357):1.798452916,Alouatta_caraya:3.181346231):1.536517229):6.356741779,((Ateles_geoffroyi:2.35930451,(Ateles_hybridus:1.3346153,Ateles_belzebuth:1.334615311):1.024689157):5.164301101,((Lagothrix_lagotricha:0.8180502431,Lagothrix_poeppigii:0.8180502092):2.121116165,Brachyteles_arachnoides:2.939166419):4.584439068):3.550999603):4.944896318):1.045045327):14.25076738,(((((Macaca_sylvanus:5.237972351,((Macaca_sinica:3.563063376,((Macaca_fascicularis:2.872078962,(((Macaca_mulatta:1.396457778,(Macaca_fuscata:1.037619442,Macaca_nigra:1.037619437):0.3588383825):0.3070918557,Macaca_cyclopis:1.703549646):0.5998889981,Macaca_arctoides:2.303438636):0.5686403092):0.6031531814,((Macaca_assamensis:1.504573706,Macaca_thibetana:1.504573719):1.411728956,Macaca_radiata:2.916302614):0.5589294313):0.0878311896):0.6800948751,((Macaca_nemestrina:1.422780536,Macaca_silenus:1.422780509):1.999441824,(Macaca_tonkeana:1.728703894,Macaca_maura:1.728703862):1.693518475):0.8209357447):0.9948143787):2.311505521,((Theropithecus_gelada:4.451390611,((Papio_ursinus:1.317971136,Papio_cynocephalus:1.317971136):0.7061627504,(Papio_papio:1.552831225,(Papio_anubis:1.072260208,Papio_hamadryas:1.07226018):0.4805710175):0.4713026754):2.427256802):2.092832835,(Mandrillus_sphinx:4.079459848,(Cercocebus_atys:2.383879806,(Lophocebus_ougandae:0.2180830922,Cercocebus_torquatus:0.2180830922):2.165797):1.695580056):2.464763628):1.005254547):1.773568178,((((Erythrocebus_patas:4.862864922,(Cercopithecus_solatus:2.084195591,Chlorocebus_pygerythrus:2.084195542):2.778669484):0.7200368163,Cercopithecus_petaurista:5.582901831):0.4174467646,(Cercopithecus_diana:4.792306391,Cercopithecus_campbelli:4.792306592):1.208042047):1.683478344,Miopithecus_talapoin:7.683826911):1.639219296):3.474883767,(((((Semnopithecus_schistaceus:2.077406301,T

rachypithecus_obscurus:2.077406308):0.8423516303,Trachypithecus_cristatus:2.91975797):3.210233424,Semnopithecus_entellus:6.1299915)
:1.277128545,Presbytis_thomasi:7.407119813):0.5453350979,((Pygathrix_nemaeus:5.417253627,Nasalis_larvatus:5.41725364):0.9834157279,
Rhinopithecus_bieti:6.400669234):1.551785703):4.845474969):7.925439124,((Gorilla_gorilla:1.083180414,Gorilla_beringei:1.083180414):4.51
4312963,(Pan_troglodytes:1.355478226,Pan_paniscus:1.355478226):4.242015071):15.12587564):10.59194493):35.73064897);
